# The evolutionary arms race between transposable elements and piRNAs in *Drosophila melanogaster*

**DOI:** 10.1186/s12862-020-1580-3

**Published:** 2020-01-28

**Authors:** Shiqi Luo, Hong Zhang, Yuange Duan, Xinmin Yao, Andrew G. Clark, Jian Lu

**Affiliations:** 1 State Key Laboratory of Protein and Plant Gene Research, Center for Bioinformatics, College of Life Sciences and Peking-Tsinghua Center for Life Sciences, Peking University, Beijing, 100871 China; 2 College of Plant Protection, Beijing Advanced Innovation Center for Food Nutrition and Human Health, China Agricultural University, Beijing, 100193 China; 3 Academy for Advanced Interdisciplinary Studies, Peking University, Beijing, 100871 China; 4 Department of Molecular Biology and Genetics, Cornell University, Ithaca, NY 14853 USA

**Keywords:** Transposable element, piRNA, Arms race, Co-evolution, *Drosophila melanogaster*

## Abstract

**Background:**

The *piwi*-interacting RNAs (piRNAs) are small non-coding RNAs that specifically repress transposable elements (TEs) in the germline of *Drosophila*. Despite our expanding understanding of TE:piRNA interaction, whether there is an evolutionary arms race between TEs and piRNAs was unclear.

**Results:**

Here, we studied the population genomics of TEs and piRNAs in the worldwide strains of *D. melanogaster*. By conducting a correlation analysis between TE contents and the abundance of piRNAs from ovaries of representative strains of *D. melanogaster*, we find positive correlations between TEs and piRNAs in six TE families. Our simulations further highlight that TE activities and the strength of purifying selection against TEs are important factors shaping the interactions between TEs and piRNAs. Our studies also suggest that the de novo generation of piRNAs is an important mechanism to repress the newly invaded TEs.

**Conclusions:**

Our results revealed the existence of an evolutionary arms race between the copy numbers of TEs and the abundance of antisense piRNAs at the population level. Although the interactions between TEs and piRNAs are complex and many factors should be considered to impact their interaction dynamics, our results suggest the emergence, repression specificity and strength of piRNAs on TEs should be considered in studying the landscapes of TE insertions in *Drosophila*. These results deepen our understanding of the interactions between piRNAs and TEs, and also provide novel insights into the nature of genomic conflicts of other forms.

## Background

The conflicts between two competing species could continuously impose selective pressures on each other, potentially causing an evolutionary arms race [1, 2]. The “attack-defense” arms race, in which offensive adaptation in one species is countered by defensive adaptation in the other species (such as the predator-prey or the parasite-host asymmetry), could lead to three possible scenarios: 1) one side wins and drives the other to extinction, 2) one side reaches an optimum while displacing the other from its optimum; or, 3) the race may persist in an endless cycle [3]. Intra-genomic conflicts, the antagonistic interactions between DNA sequences (or their products) within the genome of the same species, can also lead to an evolutionary arms race at the molecular level [4–7]. Among various systems of genomic conflicts, an important form is the interaction between transposable elements (TEs) and the host genomes [8, 9]. TEs are selfish genetic elements that are generally detrimental to the host organism [10–17]. The abundance of TEs varies dramatically across eukaryotes [10], ranging from ~ 1% [18] to more than 80% of the genome [19]. TEs impose a high fitness cost on the host organism through three possible mechanisms: 1) disrupting coding or regulatory regions of genes [20–24]; 2) eroding cellular energy and resources [25, 26]; or 3) nucleating ectopic recombination to induce chromosomal rearrangements [27–31].

*Drosophila melanogaster* provides a good system to study the molecular mechanisms and evolutionary dynamics of TEs [29, 32–35]. TEs make up at least 5% of the euchromatic genome of *D. melanogaster* [36–41], and approximately 50–80% of mutations arising in *D. melanogaster* can be attributed to TE insertions [21, 42]. Although TE insertions in *Drosophila* have frequently been associated with adaptive evolution [43–47], TEs are overall selected against in *Drosophila* [20–30, 47–50]. PIWI-interacting RNAs (piRNAs), a class of small RNAs that specifically repress TEs expressed in animal germlines, were first discovered in *Drosophila*. The discovery of piRNAs has considerably deepened our understanding of the molecular mechanisms underlying the interactions between TEs and the host organisms [51–59]. The biogenesis and functional mechanisms of piRNAs exhibit features that are distinct from miRNAs and endogenous siRNAs [56, 60–67]. In *Drosophila*, piRNAs are small RNAs of approximately 23–29 nucleotides in length bound by Piwi-class Argonaute proteins (PIWI, AUB, and AGO3). Mature piRNAs are processed from piRNA precursors, which are usually transcribed from degenerated copies of TEs that form large clusters in heterochromatic regions of the *Drosophila* genome (called “piRNA clusters”) [56, 68–76]. Mature piRNAs repress their target mRNAs through a positive feedback loop called the “Ping-Pong cycle”, in which primary and secondary piRNAs alternatively cleave mRNAs of TEs [56, 77, 78].

The piRNA pathway well explains the molecular mechanisms underlying the *P-M* system of hybrid dysgenesis in *Drosophila* [61, 79]. The *P-*element is a DNA transposon that invaded *D. melanogaster* from *D. wilistoni* by horizontal transfer within the last 100 years, and the *P*-element is still polymorphic in the populations of *D. melanogaster* [80–82]. Although *P-*elements replicate in a “cut-and-paste” manner, they increase their copy number in the genomes through homologous repair from sister strands [83, 84]. Notably, many strains of *D. melanogaster* have generated piRNAs that specifically repress *P-*elements despite the recent insertions [61]. Since piRNAs are maternally deposited into the eggs and early embryos [56, 85–87], the maternal deposition of *P*-element corresponding piRNAs neatly explains the reciprocal cross difference in hybrid dysgenesis between *P* and *M* strains of *D. melanogaster* [61]. Besides, the piRNA machinery also provides novel insights into other long-lasting evolutionary phenomena in *Drosophila,* such as the TE-repressing effects of the *flamenco* locus [56, 88], and the *I-R* system of hybrid dysgenesis [89, 90].

Novel TE insertions are pervasive and highly variable in *Drosophila*. The host organisms could quickly develop novel piRNAs that specifically repress the novel invaded TEs through distinct mechanisms. For example, previous studies have demonstrated that the de novo production of piRNAs repressing *P*-elements could be achieved very rapidly in *D. melanogaster* after *P*-element invasions [79, 91–93]. In addition, de novo piRNAs can also be generated in the flanking regions of novel inserted sites of other TE families [71, 94–96]. Besides being generated from de novo sites, piRNAs can also be produced from the pre-existing piRNA clusters after a novel TE invades into that cluster. For example, in *D. simulans,* piRNAs were quickly produced to suppress the *P*-elements that were inserted into pre-existing piRNA clusters [97]. Also, after introducing the *Penelope* TE into *D. melanogaster*, piRNAs were generated to suppress *Penelope* after this TE jumped into a pre-existing piRNA cluster [98]. Nevertheless, it yet remains unclear which of the two mechanisms is the dominant mechanism to produce novel piRNAs that suppress a novel invading TE.

Given the importance of piRNAs in repressing TEs, several groups have studied the evolutionary dynamics of TE/piRNA interactions using *Drosophila* as the model [95, 99–101]. Previously, we (Lu & Clark) modeled the population dynamics of piRNAs and TEs in a population genetics framework [99]. Our results suggest that piRNAs can significantly reduce the fitness cost of TEs, and that TE insertions that generate piRNAs are favored by natural selection [99]. Similar conclusions were drawn by other studies as well [102, 103]. Since piRNAs suppress activities of the target TEs, one might intuitively expect to observe a negative correlation between the copy numbers/activities of TEs and piRNAs at the population level. However, other studies have shown that there might be evolutionary arms race between TEs and TE-derived piRNAs from different aspects. First, TE-derived piRNA abundance tends to be positively correlated with TE expression in individual strains of *D. melanogaster* and *D. simulans* [101, 104]. Second, it was shown that although the signal of ping-pong amplification and piRNA cluster representation affect TE-derived piRNA abundance in a strain, the level of piRNA targeting is rapidly lost for inactive TEs in that strain [101]. Third, TE expression is negatively correlated with activities of piRNA pathway genes at the population level [104], and intriguingly, the effector proteins in piRNA machinery also show strong signatures of adaptive evolution [105–107]. These results suggest that the genes in the piRNA pathway machinery might be involved in the arms-race co-evolutionary processes between TEs and piRNAs (or the host organisms). Moreover, our previous studies also demonstrated that piRNAs may provide a shelter for TEs in the genomes since the detrimental effects of TEs are alleviated [99]. Based on these observations, here, we hypothesized the competitive interactions between TEs and piRNAs could lead to an arms race because of the detrimental effects imposed by TEs and the selective advantage conferred by piRNAs in repressing TEs. Previously, Song et al. sequenced small RNAs in ovaries of 16 *D. melanogaster* strains from the DGRP project [108, 109]. However, they did not find a simple linear correlation between the global piRNA expression and novel TE insertions (the polymorphic insertions) across the 16 DGRP strains [95]. Here, we aimed to test the TE/piRNA evolutionary arms race hypothesis with another population genomic dataset of *D. melanogaster*. Under the piRNA:TE evolutionary arms race scenario, we expect to observe a positive correlation between TE content and piRNA abundance among different strains.

In this study, we first examined the abundance of TEs and their respective piRNAs in the worldwide Global Diversity Lines (GDL) of *D. melanogaster* [110]. We found the novel TE insertions frequently induced de novo piRNA generation from the flanking regions of the insertion sites. We then conducted correlation analysis between TE contents and the abundance of piRNAs from ovaries of 26 representative strains of *D. melanogaster*, and detected significantly positive correlations for six TE families. We also conducted forward simulations with the parameters optimized for *D. melanogaster* to investigate the factors influencing the evolutionary arms race between TEs and piRNAs.

## Results and discussion

### The contents of TEs vary across populations of *D. melanogaster*

**Fig. 1:**
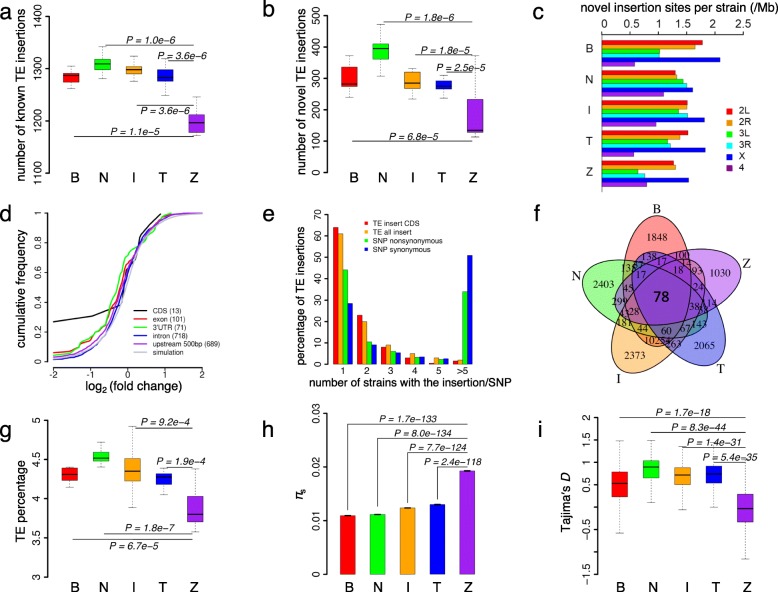
The contents and polymorphisms of TE insertions in *D. melanogaster* from the Global Diversity Lines (GDL). The five populations are abbreviated as follows: B, Beijing (*n* = 14); I, Ithaca (*n* = 17); N, Netherland (*n* = 19); T, Tasmania (*n* = 17); Z, Zimbabwe (*n* = 14). **a** Boxplots of the numbers of known TE insertions (*y-*axis) across the five populations. The average copy number (± s.e.) in each strain is 1283.7 ± 3.3, 1297.4 ± 3.4, 1309.1 ± 3.5, 1290.5 ± 6.9 and 1204.3 ± 8.4 for the B, I, N, T, and Z population, respectively. **b** Boxplots of the numbers of novel TE insertions (*y-*axis) across the five populations. The average number (± s.e.) of novel insertions in each strain is 299.1 ± 11.1, 288.6 ± 7.1, 387.9 ± 10.3, 275.8 ± 5.0, and 171.5 ± 19.8 in the B, I, N, T, and Z population respectively. **c** Densities (insertions per Mb) of TE novel insertion sites on different chromosomes per strain in five populations. **d** Changes of gene expression caused by TE insertions in female adults. For each novel TE insertion in the genic regions in the 5 GDL strains that have transcriptome sequenced in females, we compared the expression level of the host genes in the strains that have the TE insertion vs. the strains that do not have the particular insertion. The *x-*axis is the log_2_ (fold change) of gene expression caused by a TE insertion. The *y*-axis is the cumulative probability of each insertion category. **e** Frequency spectra of novel TE insertions and SNPs from different functional categories. The *x-*axis is the number of strains that carry the particular category of TE insertions or SNPs, and the *y*-axis is the percentage of TE insertions or SNPs in each class that is segregating at that particular frequency. **f** Venn diagram of novel TE insertions across the five populations. **g** The percentages of genomic reads (*y-*axis) that are mapped to the TEs annotated in the reference genome across the five populations. **h** Barplots of *π*_s_ in 10 kb bins across the five populations. **i** Boxplots of Tajima’s *D* in 10 kb bins across the five populations. KS tests were performed to test the differences in the statistic values across populations

Empirical tabulation of the abundances of TEs and piRNAs across a series of wild-derived fly strains will serve as the initial substrate for learning about their co-evolutionary dynamics. The strains of *D. melanogaster* sequenced in the GDL project were collected from five continents (B, Beijing; N, Netherlands; I, Ithaca, New York; T, Tasmania; and Z, Zimbabwe), and these strains were sequenced at ~ 12.5× coverage [110]. For each of the 81 strains sequenced with the Illumina 100 bp paired-end protocol, we mapped the genomic shotgun reads to the reference genome of *D. melanogaster* and characterized TE insertions with two complementary methods (Methods). First, for each TE insertion annotated in the reference genomes of *D. melanogaster* (called the “known” insertions)*,* we examined whether it was present in the 81 GDL strains based on the mapping results of the flanking sequences. Among the 3544 known TE insertions that have unique boundary sequences in the reference genome, the average copy number (±s.e.) in each strain ranged from 1204.3 ± 8.4 to 1309.1 ± 3.5 in the five populations (Fig. 1a). Notably, 600 (26.8%) of the known TE insertions were not found in any GDL strain, supporting the notion that unique transposon insertions are pervasive in the populations of *D. melanogaster* [100]. As expected [31], these reference-genome-specific insertions are mainly caused by longer TEs (the length is 5088.9 ± 131.1 versus 1853.1 ± 52.0 nts of the remaining TEs in the reference genome; *P* < 10^− 10^, Kolmogorov–Smirnov test [KS test]). Second, in each GDL strain, we employed TEMP [111], which was designed to detect novel TE insertions in *Drosophila,* to systematically identify possible novel TE insertions that are not present in the reference genome of *D. melanogaster*, and we further filtered the original TEMP results based on strict criteria to remove possible false-positive results (Methods). In total, we identified 11,909 novel insertion sites of TEs that were present in the GDL strains but absent in the reference genome, and the average number of novel insertions in each strain ranges from 171 to 388 in the five populations (Fig. 1b). To assess the TEMP performance in TE detection, we compared the results obtained in the ~ 12.5× coverage of ZW155 strain versus those obtained with an independent 100× coverage paired-end re-sequencing of this same strain [110]. Of the 238 novel insertions detected in the 12.5× sequencing, 198 were independently verified using the 100× coverage re-sequencing result, yielding a call rate repeatability of 83.2%. Among the novel insertions, 61.3% of the insertions were caused by LTRs, 19.2% caused by DNA transposons and 14.6% mediated by non-LTRs.

As previously shown [112, 113], the novel TE insertion sites are significantly enriched in the X chromosome after controlling for the size differences of chromosomes (Table 1, Fig. 1c). The majority of the novel insertions occurred in introns (56.9%), followed by 3′ UTRs (5.60%), ncRNAs (3.98%), 5′ UTRs (2.37%), and CDSs (1.80%) (Additional file 1: Table S1). TE insertions often disrupt CDSs or regulatory sequences [31, 40, 46]. To explore the impact of TE insertions on the expression levels of the host genes, we examined the whole-body transcriptomes of adult females for 5 GDL strains (B12, I17, N10, T05, and ZW155) [114]. As expected [50, 95, 115], we found genes with novel TE insertions in exons, especially in CDSs, had significantly reduced expression levels (Fig. 1d) when we compared gene expression levels in the strains with a TE insertion versus the strains without that particular TE insertion. By contrast, TE insertions in introns or 500 bp upstream of the TSS (transcriptional start site) are not associated with significant changes in gene expression levels (Fig. 1d).

**Table 1:** Summary of the novel TE insertions in different chromosomes in the GDL strains

Chromosome		All novel insertions		Frequency			Frequency (TE > 5 kb)		
Name	Length	Observed	Expected	1	2–5	> 5	1	2–5	> 5
2 L	23,513,712	2279	2088.09	1486	717	76	1047	444	20
2R	25,286,936	2328	2245.55	1474	741	113	1060	447	53
3 L	28,110,227	2013	2496.27	1142	753	118	837	471	52
3R	32,079,331	2352	2848.74	1187	1009	156	884	652	69
4	1,348,131	73	119.72	55	16	2	40	10	1
X	23,542,271	2844	2090.62	1996	780	68	1374	442	30

To identify the adaptive TE insertion events that left footprints in the genomes, we calculated Tajima’s *D* [116] and Fay & Wu’s *H* [117] values in a binned window of 10 kb (Additional file 1: Figures S1 and S2) and the composite likelihood ratio (CLR) [118–120] with SweeD [121] in each local and the global population (Additional file 1: Figure S3). We identified 24 high-frequency TE insertions (present in at least 5 strains) that have flanking SNPs with *D* < − 1 and *H* < − 1 in the local or global populations (Additional file 1: Table S2), among which three TE insertions fall within the top 5% CLR distribution in the corresponding analysis, including one *412* insertion in *Dystrophin* (Additional file 1: Figure S4). These results suggest such TE insertions potentially lead to local adaption in the GDL strains.

Compared to the derived synonymous or nonsynonymous mutations (Methods), the frequency spectra of the TE insertions are significantly skewed to lower frequencies (*P* < 0.0001 in each comparison, Fisher’s exact tests; Fig. 1e), suggesting that novel insertions of TEs are overall under stronger purifying selection. Specifically, among the novel insertions of TEs, 9719 (61.9%) were detected in a single GDL strain, 537 (4.51%) were present in more than five strains, and only 78 insertions were shared among all the five populations (Fig. 1f). Accordingly, the multidimensional scaling (MDS) analysis of the known (Additional file 1: Figure S5a) and novel (Additional file 1: Figure S5b) insertions of TEs suggests that strains from the same population are well clustered. Interestingly, the Z strains, in general, have the lowest numbers of known (Fig. 1a) and novel (Fig. 1b) TE insertions. Moreover, the Z strains have significantly lower fractions of reads from TEs that are mapped on the reference genome than the other four populations (*P* < 0.0001 in each comparison, KS test, Fig. 1g). Since some TEs are absent in the reference genome of *D. melanogaster* [122] and the level of TE sequence diversity might be different in the five populations, we also mapped the genomic reads on the TE sequences annotated in *Drosophila* Genome Project (BDGP) TE dataset and RepBase Update [123] using BLAT [124] with different thresholds of mapping length and identity. We still obtained similar results despite the different mapping thresholds (Additional file 1: Figure S6). Previous studies indicate the Z population, which has a larger effective population size than the non-African populations [125–129], experienced a recent growth [130–132], and the non-African populations often experienced bottleneck after migration out of Africa [130, 132]. Consistently, the Z population in the GDL strains have significantly higher nucleotide diversity (*π*_s_) and lower Tajima’s *D* values than the N, I, B, and T populations (*P* < 10^− 16^ in each comparison, KS tests; Fig. 1h, i). Since the efficacy of natural selection is inversely influenced by the effective population size [133], purifying selection might have eliminated deleterious TE insertions more efficiently in the Z strains.

Altogether, in this study, we detected abundant TE insertions that are polymorphic in the population of *D. melanogaster*, and the Z population from Africa harbors fewer TE insertions than other populations, which might be related to the stronger purifying selection. The heterogeneity of TE insertions among strains of *D. melanogaster* enables us to test the possible evolutionary arms race between TEs and their suppressors at the population level.

### Profiling piRNAs in ovaries of 10 representative GDL strains by deep sequencing

**Fig. 2:**
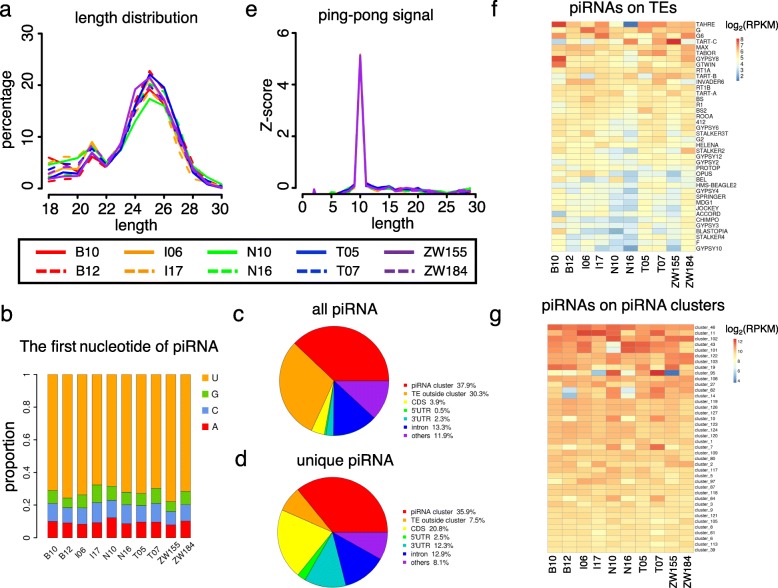
Characteristics of small RNAs sequenced in 10 GDL strains. **a** Length distribution of small RNAs that are mapped to the reference genome and TE sequences, the known miRNAs, tRNAs, rRNAs, ncRNAs and miscRNAs were removed. **b** Barplots of the fractions of the first nucleotide of piRNAs in 10 GDL strains. **c** Pie chart of the genomic locations for all mapped piRNAs. **d** Pie chart of the genomic locations for the uniquely mapped piRNAs. **e** The ping-pong signature generated between the sense and antisense piRNA reads. The *x-*axis shows the nucleotides that are overlapping between a sense and antisense piRNA. The *y-*axis is the Z-score of the overlapping length among all the possible overlapping combinations. **f** Heatmap showing the RPKM values of weighted piRNAs on TEs in 10 GDL strains. Only the top 40 TEs with the highest RPKMs are shown. **g** Heatmap showing the RPKM values of weighted piRNAs on piRNA clusters in 10 GDL strains. Only the top 40 piRNA clusters with the highest RPKMs are shown

To explore the impact of piRNA repression on the TE distributions in the GDL strains, we deep-sequenced small RNAs from ovaries of 3–5-day-old females in 10 representative GDL strains that were collected from five continents (see Additional file 1: Table S3 for sequencing statistics). We mapped the small RNAs onto the reference genome of *D. melanogaster* and TE sequences collected from BDGP TE dataset and RepBase Update [123] (Methods). In case a small RNA read was mapped to multiple locations, it was equally split across these locations. After removing reads that mapped to rRNAs, tRNAs, miscRNAs, ncRNAs and miRNAs, the remaining small RNAs that mapped to the reference genome show a major peak at 25 nt (ranging from 23 to 29 nts) and a minor peak at 21 nt (ranging from 20 to 22 nts), which are typical lengths of piRNAs and endogenous siRNAs, respectively (Fig. 2a). As expected [56, 86, 111, 134, 135], ~ 72.1% of the piRNA-like reads (23–29 nt) in our study had uridine in the first position of the 5′-end (referred as “1 U”, Fig. 2b). Overall, 45.6–51.7% of all the mapped 23–29 nt piRNA-like reads were from TEs, suggesting TEs are the major source for piRNAs. Although 34.8–39.7% of all the mapped piRNA-like reads were located in previously identified piRNA clusters [56, 86, 134, 135], 26.0–31.8% of them mapped onto TEs outside the known clusters (Fig. 2c). If we only considered the piRNA-like reads that were uniquely mapped to the genome and TE reference sequences, we found 25.8–43.6% of the piRNA reads were mapped to the known piRNA clusters, and 3.7–9.2% of them were mapped to TEs outside the piRNA clusters (Fig. 2d). These results suggest some piRNAs are either produced from novel piRNA clusters or through a piRNA-cluster-independent approach. In the “Ping-Pong” cycle of piRNA suppression and amplification, a sense-strand piRNA that is bound by Ago3 recognizes a complementary piRNA transcript and Ago3 cleaves the target at the site corresponding to the 10th nucleotide of the loaded piRNA, generating a new antisense piRNA that is bound by Aub. Then the Aub-loaded piRNA recognizes and cleaves a complementary TE transcript, generating a new piRNA identical to the initial Ago3-loaded piRNA [56, 78, 86, 134, 135]. The 10 nt overlap between an Ago3-loaded sense piRNA and Aub-loaded antisense piRNA is a hallmark for piRNA biogenesis and functioning in the presence of the active target TE. In each sample, we detected significant “Ping-Pong” signals in all the piRNA-like reads (Fig. 2e), highlighting that our sequencing results have well captured the interactions between piRNAs and active TEs.

Among various TE families, the reference sequences of *TAHRE, G, G6, TART-C,* and *MAX* have the highest density of piRNAs (Fig. 2f). For the 29 TE families whose reference sequences have the mean piRNAs density > 20 RPKM among strains, the median coefficients of variation (*cv*, defined as sd/mean of expression across strains) is 0.38, with piRNAs on the sequences of *TART-C*, *GYPSY8*, *GTWIN, OPUS* and *BEL* families most variable across the 10 GDL strains. For the 56 known piRNA clusters that have piRNA density > 20 RPKM, the *cv* value ranged from 0.054 to 0.74, with a median value of 0.20, suggesting the piRNAs generated in these clusters are also variable across strains (Fig. 2g).

**Fig. 3:**
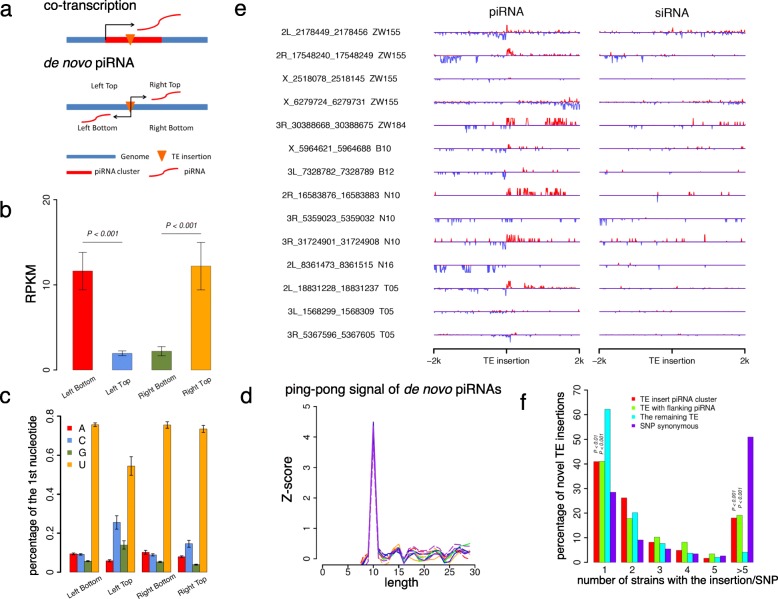
Generation of de novo piRNAs in the flanking regions of novel TE insertions. **a** A schematic diagram illustrating the two hypotheses of how novel piRNAs are induced from TE insertions. The first mechanism is that a TE jumps into a pre-existing piRNA locus so that novel piRNAs are generated by co-transcription of the established piRNA precursor. The second mechanism is that de novo piRNAs are generated in the flanking region of novel TE insertions. **b** Barplots showing the RPKMs of de novo piRNAs generated in the flanking region (upstream and downstream 2 Kb) of novel TE insertions. The de novo piRNAs are generated with strong strand-asymmetric distributions. KS tests were performed to test the differences in the RPKM values. **c** Barplots of the fractions of the first nucleotide of de novo piRNAs generated in the flanking region (upstream and downstream 2 Kb) of novel TE insertions. **d** The ping-pong signature of de novo piRNAs generated in the flanking region (upstream and downstream 2 Kb) of novel TE insertions in 10 GDL strains. The color key for the strains is the same as shown in Fig. 2a. **e** Examples of de novo piRNAs and siRNAs generated from the flanking region of *P-*element insertion in 10 GDL strains. The sense-strand small RNAs are plotted in red, and the anti-sense small RNAs are plotted in blue. **f** Frequencies of novel TE insertions and SNPs. The *x-*axis is the number of strains that carry the particular category of TE insertions or SNPs, and the *y*-axis is the percentage of TE insertions or SNPs in each class that is segregating at that particular frequency. The TE insertions in piRNA clusters or with de novo piRNAs are segregating at higher frequencies. Fisher’s exact tests were performed to test the differences in the RPKM values

Besides being generated from de novo sites, piRNAs can also be produced from the pre-existing piRNA clusters after a novel TE invades into that cluster (Fig. 3a). However, it yet remains unclear which of the two mechanisms is the dominant mechanism to produce novel piRNAs that suppress a novel invading TE. We found 18 novel TE insertions in the known piRNA clusters in the 10 GDL strains. For example, the X-linked *flamenco* piRNA cluster harbors the largest number of novel TE insertions in the 10 GDL strains (Five novel TE insertions regions were observed in this locus, Additional file 1: Figure S7), followed by the piRNA cluster *42AB* on 2R, which hosts three novel TE insertions (Additional file 1: Figure S8). By contrast, we found 343 out of 2632 (13.0%) novel TE insertions that have signals of de novo 23–29 nt piRNAs in at least one strain with the uniquely mapped reads (Table 2). Consistent with previous observations [94, 95], the de novo piRNAs are generated with strong strand-asymmetric distributions: the majority of the piRNAs in the left flank are in the antisense strands while most of the piRNAs in the right flank are generated in the sense strands (Fig. 3b and Additional file 1: Figure S9). The piRNAs in the flanking regions are also enriched in 1 U signatures (Fig. 3c) and show the typical ping-pong signature (Fig. 3d). Notably, we frequently detected endogenous siRNAs in those regions flanking the TE insertion (Additional file 1: Figure S10, an example of *P-*element is displayed in Fig. 3e), although it is yet unclear whether such siRNAs are involved in the induction of the de novo piRNAs.

**Table 2:** Novel TE insertions in the 10 strains that have piRNAs (23–29 nt) uniquely mapped to the regions 2 kb up- or down-stream of the inserted sites

Strain	Novel TE insertion regions	Novel TE insertion regions with unique piRNAs
B10	336	54 (16.1%)
B12	292	39 (13.4%)
I06	260	22 (8.5%)
I17	255	26 (10.2%)
N10	455	33 (7.3%)
N16	306	42 (13.7%)
T05	251	31 (12.4%)
T07	272	24 (8.8%)
ZW155	248	42 (16.9%)
ZW184	370	49 (13.2%)

Our previous results suggest that novel insertions in the piRNA clusters are favored by natural selection, since they generate piRNAs that repress active TEs [99]. Accordingly, in the GDL strains the novel insertions in the piRNA clusters are overall segregating at higher frequencies than the remaining novel insertions (Fig. 3f). Interestingly, the TE insertions that have de novo piRNA production signals in the flanking regions are also segregating at higher frequencies than the remaining TE insertions (22.6 and 6.17% of the TE insertions are segregating in at least 5 strains for the former and latter classes, respectively; *P* < 0.001, Fisher’s exact test; Fig. 3f). It is possible that these novel insertions might be advantageous, since the de novo piRNAs might repress other detrimental TEs through trans-acting effects. Nevertheless, we could not exclude the possibility that the de novo piRNAs generated by a novel insertion will alleviate the deleterious effects of the inserted TE itself so that it is under relaxed selective constraints.

Together, our results suggest that de novo induction is more prevalent than piRNA cluster trapping for novel piRNA biogenesis in natural populations of *D. melanogaster*. As expected, novel TE insertions with piRNA cluster trapping and de novo piRNA generation tend to segregate at higher frequencies in the populations. Importantly, the abundance of piRNAs is variable in the ovaries of different *D. melanogaster* strains, raising the possibility that the variation in piRNAs might be coupled to the variation in TEs.

### Relationship between piRNA abundances and TE copy numbers across strains of *D. melanogaster*

To test the evolutionary arms race between piRNAs and TEs at the population level, we examined the relationship between piRNA abundances and the total TE copy numbers across the 10 representative GDL strains of *D. melanogaster*. In each strain, we predicted the target TEs of the piRNAs by requiring the perfect match between the 2–11 positions of piRNAs and the target sequences (Methods). For a reference TE sequence, we calculated the density of piRNAs that putatively target that TE. In case a piRNA targets multiple TE reference sequences, it was equally split and assigned to all the predicted targets (Methods). Notably, the length of a TE is significantly positively correlated with the weighted abundance of piRNAs targeting that TE (Additional file 1: Figure S11), suggesting longer TEs which are in general more deleterious [31] are also more likely targeted by piRNAs. Across the 10 GDL strains of *D. melanogaster,* only *P*-element out of the 105 tested TE families showed a significantly positive Spearman’s correlation between TE DNA copy numbers and the weighted abundances of antisense piRNAs after multiple testing correction (adjusted *P* < 0.05 was used as cutoffs; Additional file 2: Table S4).

**Fig. 4:**
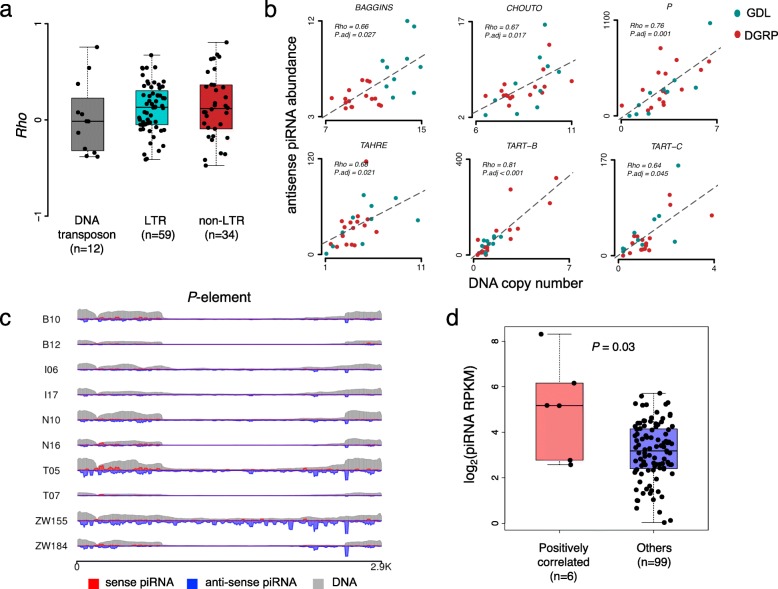
Correlations between TE DNA copy number and antisense piRNA abundance. **a** Boxplots of Spearman’s correlation coefficients (*Rho*) values between TE DNA copy number and antisense piRNA abundance in DNA transposons (*n* = 12), LTR (*n* = 59), and non-LTR (*n* = 34) families. **b** Scatter plots displaying the TE DNA copy number and antisense piRNA abundance (RPKM) for representative TE families. Dots in cyan represent the GDL strains, and dots in red represent the DGRP strains. The Spearman’s *Rho* and adjusted *P* values are shown. **c** Sequencing coverage of DNA and piRNA along *P-*element in 10 GDL strains. Sense piRNAs are shown in red; antisense piRNAs are shown in blue; and DNA is shown in grey. **d** Boxplots of antisense piRNA density between TE families, which showed significantly positive Spearman’s correlation between TE copy number and antisense piRNA abundance (*n* = 6) and other TE families (*n* = 99)

A previous study [95] has sequenced small RNAs in ovaries of 16 *D. melanogaster* strains from the DGRP project [108, 109]. Similar to our results with the 10 GDL strains, that study also did not detect significant correlations between TE insertions and piRNAs in 16 *D. melanogaster* strains after correcting for multiple testing [95]. To increase the statistical power of the correlation analysis, we combined the data from both sources and conducted the correlation analyses. The correlations between TE DNA copy numbers and antisense piRNA densities tended to mixed across the 26 strains of *D. melanogaster* (the Spearman’s *Rho* value was positive for 65 families and negative for 40 families, Additional file 2: Table S4). Of note, we did not observe significant differences in *Rho* values among DNA transposons, LTR, and non-LTR TE families (Fig. 4a). However, we found significantly positive Spearman’s correlations (adjusted *P* < 0.05) between TEs and antisense piRNAs for six TE families, among which five were retrotransposons (*CHOUTO* is LTR, and *BAGGINS*, *TAHER*, *TART-B*, *TART-C* are non-LTRs), and *P*-element was DNA transposon (Fig. 4b). Thus, increasing the sample size in future studies will deepen our understanding of the evolutionary arms race between TEs and piRNAs at the population level.

The complete *P-*element (2907 bp in length) encodes a functional transposase and is autonomous. However, most TE sequences from the *P-*element family are internally deleted and are non-autonomous [136]. Accordingly, our genome alignments of the shotgun Illumina reads revealed more reads that mapped to the ends of the complete *P-*element, suggesting the widespread existence of the defective *P*-element in the GDL strains (Fig. 4c). By contrast, only a small fraction of the *P-*element fragments is full-length (Fig. 4c). We detected the *P*-element insertions in all five populations, with the median insertion number of 13.5, 12, 21, 13, and 10 for the B, I, N, T, and Z population, respectively. In total, we detected 133 insertions of *P-*element in these 10 GDL strains, and found de novo piRNAs flanking the *P-*element for 14 of these insertions (Fig. 3e). The *P-*element-derived piRNAs were mainly located in the 5′ and 3′ ends of *P-*element and their abundance varied dramatically across the 10 GDL strains (Fig. 4c). The copy number of the active part (position 819–2527) of the full-length *P-*element was significantly positively correlated with the abundance of antisense piRNAs in ovaries of the 26 strains of *D. melanogaster* (Spearman’s *Rho* = 0.76, *P* = 1.41 × 10^− 3^ in the correlation analysis; Fig. 4b). These results suggest the existence of an evolutionary arms race between *P*-elements and piRNAs in the populations of *D. melanogaster*.

There are two different piRNA pathways in the germline and somatic cells of the gonads of *Drosophila* [86, 137]. In the somatic ovarian follicle cells, the piRNAs from *flamenco* locus are loaded on Piwi and mainly target TEs from the *gypsy* family, while the Ago3-dependent Ping-Pong cycle primarily occurs in the germline. Based on the Ping-Pong signals and Piwi-binding patterns, TEs were classified as germline-specific, somatic and intermediate groups [86, 137]. Among the six TE families that show positive correlations between TE DNA copy numbers and antisense piRNA densities, *BAGGINS, TART-B, TART-C*, and *TAHER* belong to the germline-specific group in which piRNAs showed salient ping-pong signals. Moreover, we also found TEs of the six families overall have a significantly higher density of antisense piRNAs than the remaining 99 TE families (*P* = 0.03, Fig. 4d), affirming the thesis that the observed evolutionary arms race is caused by the tight interaction between TEs and piRNAs.

Altogether, here we combined data from two sources and detected significantly positive Spearman’s correlations between TEs and antisense piRNAs for six TE families. For the remaining TE families that we did not detect statistically significant correlations, it is possible that the limited dataset (26 strains were used) or our methods lacked the power in detecting the true signals, and this does not necessarily suggest that evolutionary arms race does not exist in those TE families. TEs of different families often vary in many aspects, such as the preferences of insertion sites, the invasion history, and replication rates [113, 138], all of which might affect the relationships between TE and piRNA abundances. Therefore, more factors and more complex (or specific) models need to be considered in studying the arms race between TEs and piRNAs.

### The model of TE:piRNA interactions

**Fig. 5:**
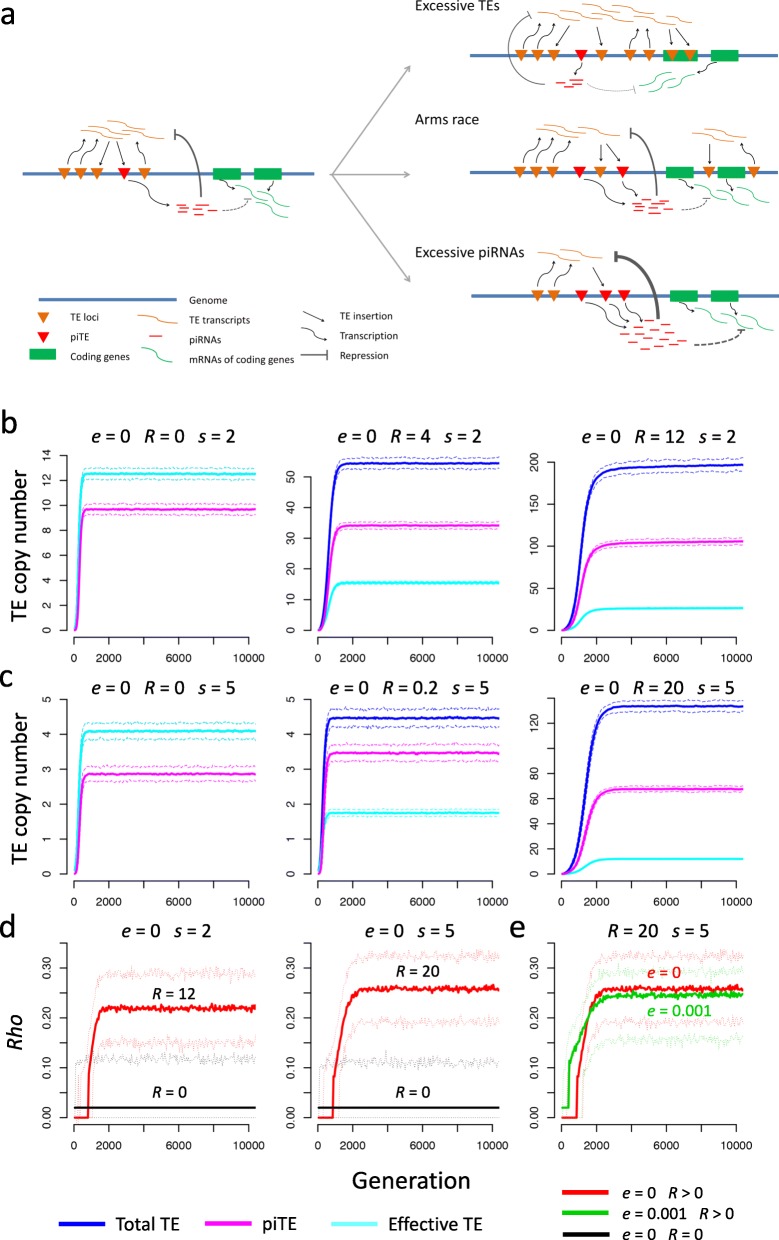
The evolutionary arms race between TEs and piRNAs revealed by simulations. **a** A schematic diagram illustrating the process and consequence of TE:piRNA interactions. Three possible consequences of TE:piRNA interactions depend on TE replication rate, the repressive strength of piRNAs on TEs, and the strength of purifying selection against TEs: 1) Excessive TEs. When TE replication rate is high and the repressive strength of piRNA is weak (TEs jumping into piRNA cluster and become piRT producing piRNAs), TEs soon become excessive in the genome, disrupt coding genes and have detrimental effects on the genome. 2) Arms race. When more piRTs produce more piRNAs and have stronger repression on TE, TE replication rate becomes lower and less TE exists in the genome, but the piRNA also alleviate detrimental effects of TEs on the genome. 3) Excessive piRNAs. If piRNA repression is very strong, TE activity becomes quite low and hardly jumps in the genome. Note that excessive dosage of piRNAs might cause off-target effects on the normal mRNAs and hence reduce the fitness of the host organism (dashed lines). The width of the lines represents the repression strength of piRNAs. **b-c** The numbers (*y*-axis) of TEs (blue), piTEs (pink), effective TEs (cyan) accumulated in one chromosome along with the generations (*x*-axis) in the simulations. Under the same selection scaling factor (*s* = 2 for **b** and *s* = 5 for **c**), higher numbers of TEs, piTEs, and the effective TEs carried by one chromosome were observed when the repressiveness of piRNAs (*R*) on TEs gets stronger. **d** Stronger repression of piRNA on the activities of TEs cause a positive correlation between piRNAs and TEs. The thick red lines are the mean Spearman’s *Rho* (*y*-axis) between the abundance of piRNAs and TEs along generations (*x*-axis) in the simulations under *R* = 12 (left) or *R* = 20 (right). The thin dashed red lines are the 2.5 to 97.5% quantiles obtained in simulations. The black lines are Spearman’s *Rho* under *R* = 0. Since in both cases, the median (thick black) and the 2.5% (thin black) quantiles are both zero, and the 97.5% (thin black) quantile is displayed. **e** Escaping of TEs from piRNA repression (*e* = 0.001, green compared with *e* = 0, red) decreases the positive correlation between the copy numbers of TEs and matched piRNAs. In all of these simulations, the following parameters are used: *u* = 0.03, *N*_*e*_ = 5000, *d* = 0.003, *i* = 0.001, *r* = 10^− 8^, *p* = 0.5, *a* = 10^− 3^, *b* = 5 × 10^− 4^, *f* = 0.2, *e* = 0 in **b**-**d**. The *R* and *s* values are displayed on each panel. The correlation was calculated in 1000 sampled chromosomes that have at least one TE from the populations. All simulations were performed for 200 replicates

In order to explore how the observations of variation in TE and piRNA abundances may impact their coevolution, we conducted forward simulations of TE:piRNA interaction dynamics in populations of *D. melanogaster* using procedures similar to those we described previously [99]. Briefly, we assumed: 1) a diploid, panmictic, constant-sized (effective population size *N*_*e*_) Wright-Fisher population (non-overlapping generations); 2) the chromosome size is 100 Mb and the homogeneous recombination rate per nucleotide is *r*; 3) in each generation the probability that a TE inserts into a new site and becomes a piRNA-generating site is *f,* 4) the duplication rate of a TE or piRNA locus per generation is *d*; 5) the probability that a TE is excised or inactivated is *i*; 6) the probability that a TE mutates to a new subtype and escapes the repression effect of a piRNA is *e*; and 7) only the TE that does not generate piRNAs can replicate; a TE of subtype *j* that is not targeted by any matching piRNA replicates at rate *u* per element per generation; and a TE of *x*_*j*_ sites that is targeted by the matched piRNAs with *y*_*j*_ sites replicates at a rate $u/\left(1+R.\frac{y_j}{x_j}\right)$, where *R* is a constant representing piRNA repression efficiency. Note that in our model TEs and piRNA loci are on the same scale, piRNAs repress TEs with “enzymatic” kinetics and in a dosage-dependent manner, and the activities of TEs in each individual are determined by the abundance of matched piRNAs as well as the numbers of TEs which compete with each other for the matched piRNAs in that individual. We also considered sequence divergence between TE copies, and the piRNAs only repress TEs of the same subtype. We assumed TEs overall imposed fitness cost in a negative epistatic manner [99, 139, 140]. Specifically, the fitness of each individual in each generation is modeled by an exponential quadratic function, $w={e}^{-s.a.n-\frac{1}{2}s.b.{n}^2+p.\left(-s.a.m-\frac{1}{2}s.b.{m}^2\right)}$, where *a* and *b* are constants, *s* is a scaling constant, *n* is the effective number of active TEs, with $n=\sum \limits_{j=1}^k{x}_j/\left(1+R.{y}_j/{x}_j\right)$ and *x*_*j*_ and *y*_*j*_ being the copy numbers of TE and piRNA sites for a TE subtype *j* in that individual; *m* is the number of excessive piRNAs, with $m=\max \left(0,\sum \limits_{j=1}^k{y}_j-{\mathrm{x}}_j\right)$, and *p* is the penalty coefficient of excessive piRNAs on the fitness of the host organism. Note here we assumed excessive dosage of piRNAs might cause off-target effects on the normal transcriptomes and hence reduce the fitness of the host organism [107]. Moreover, although our model is designed for the “copy-and-paste” replication of retrotransposons, it is also applicable to DNA transposons which increase their copy numbers in the genome through the homologous repair from sister strands [83, 84]. piRNAs repress TE activities by degrading mRNAs [56] or suppressing TE transcription through mediating heterochromatin formation [135, 141–143]. Since it is still challenging to model the piRNA-mediated suppressive effect on target TE transcription quantitatively, here we only considered the repressive effects of piRNAs by degrading target mRNAs. A scheme of the TE:piRNA interaction in our model is presented in Fig. 5a.

To expedite the simulations, the parameters optimized for *D. melanogaster* were scaled by 100, as previously described [99] (see the legend of Fig. 5 for details). The different parameter settings and combinations were performed in 200 replicates. The simulations were initiated by assuming 10% of the individuals carrying the one TE randomly (Methods).

### The evolutionary arms race between TEs and piRNAs revealed by simulations

To investigate the relative contributions of the factors in shaping the dynamics of TEs and piRNAs, we fixed the scaled parameters such as the replication rate (*u* = 0.03), the effective population size (*N*_*e*_ = 5000), the duplication rate (*d* = 0.003), the excision/inactivation rate (*i* = 0.001), the recombination rate (*r* = 10^− 8^ per nucleotide), the escape rate (*e* = 0), the penalty of excessive piRNAs (*p* = 0.5), the constants *a* = 10^− 3^ and *b* = 5 × 10^− 4^. Although the size of the piRNA loci accounts for ~ 5% of the euchromatin of *D. melanogaster* [56], many de novo piRNAs are generated outside the piRNA loci after a novel TE insertion [71, 94–96]. Therefore, we arbitrarily set *f*, the probability that a newly inserted TE is a piRNA-generation site, at 0.05 or 0.2 in our simulations. We varied the piRNA repression efficiency parameter *R* (0, 0.2, 4, 12, and 20) and the selection scaling factor *s* (0.5, 2, 5, 10, and 15) to explore the relationships between TEs and piRNAs in the populations.

Since the fitness cost of TEs has an exponential quadratic function [139, 140], TEs accumulate rapidly in the population and ultimately cause the extinction of the host organism if natural selection is weak (*s* = 0.5, Additional file 1: Figure S12). By contrast, when the selection is very strong (*s* = 20), TEs are quickly removed from the population (Additional file 1: Figure S12). The outcomes of these two scenarios are very similar to the “one-side wins” scenario of inter-species evolutionary arms races, except that TEs are part of the host genomes. As expected under the traditional replication-selection model [20, 27–29], the numbers of TEs carried by one chromosome reaches equilibrium in the population when the intensity of natural selection is intermediate (*s* = 2, Fig. 5b; *s* = 5, Fig. 5c). Notably, the dynamics of piRNA copy number carried by one chromosome are similar to the dynamics of TEs located on the same chromosome (Fig. 5b, c). This is not surprising since in our simulations the biogenesis of piRNAs is dependent on the abundance of TEs.

To investigate whether piRNA-mediated repression of TE activities would generate a positive correlation between piRNAs and TEs, in the simulations we varied the *R* parameter, which reflects the effectiveness of piRNA repression on the activities of TEs, while keeping the other parameters fixed. At *R* = 0, when we sampled 1000 chromosomes that have at least one TE from the populations to calculate the correlation between TEs and piRNAs, we found only very weak positive correlation between the numbers of TEs and piRNAs located on the same chromosome (the median value Pearson’s *r* is 0, Fig. 5d). These results suggest that although piRNAs depend on TE insertions in biogenesis, this alone would not produce a strong positive correlation between the numbers of piRNAs and TEs accumulated in each chromosome if piRNAs do not repress TEs effectively. However, when *R* is increased, the correlation coefficient between TEs and piRNAs significantly increases after 1000 generations in the simulations (*R* = 12*, s* = 2; *R* = 2*0, s* = 5; Fig. 5d). These results indicate that stronger repression of TEs by piRNAs would yield a stronger positive correlation between TEs and piRNAs, since the deleterious effects of TEs would be alleviated by piRNA repression. Since mutations in TE sequences might cause a TE to escape the repression mediated by piRNAs, we also set *e* = 0.001 to examine the extent to which TE escaping from piRNA repression would affect the correlation. Although we still observed a significant positive correlation between the copy numbers of TEs and matched piRNAs (green, Fig. 5e), the correlation coefficient is smaller than that obtained with *e* = 0 (red, Fig. 5e). Therefore, mutations in TE target sites could potentially weaken the positive correlation between TEs and piRNAs. All the above results were obtained under the assumption that the probability that the insertion site of a novel TE is a piRNA-generating locus (*f*) is 0.2. To examine the extent to which the parameter *f* affects the population dynamics of TEs and piRNAs, we also set *f* = 0.05. If the repressiveness of piRNAs on TEs is strong (*R* = 20), we obtained very similar patterns when we set *f* = 0.2 or *f* = 0.05 (Additional file 1: Figure S13). In summary, our simulations suggest that three parameters could affect outcomes of the TE:piRNA interactions. First, the strength of natural selection is important: weak selective pressures would cause TEs to accumulate in the genomes and ultimately cause the extinction of the organisms, whereas strong natural selection would result in elimination of TEs from the population. Second, the repressiveness of piRNAs on TEs affects the arms race patterns. Third, the escaping rate of TEs from piRNA-mediated suppression would decrease the positive correlation between TEs and piRNAs.

In summary, our results suggest that if TEs can persist in the population in the long-run, the interactions between TEs and piRNAs could lead to an evolutionary arms race.

## Conclusions

piRNAs repress target TE activities by degrading mRNAs or inhibiting TE transcription [135, 141–143]. Besides piRNAs, many epigenetic factors affecting the transcription of the piRNA clusters, such as the epigenetic modifications of chromatin states [96, 144] and the interactions between the Rhino complex with the H3K9me3-marked chromatin [70, 71]. Moreover, the piRNA-mediated spread of heterochromatin from TEs into neighboring genes might disrupt the function of those genes and cause deleterious effects [115]. In this study, we only considered the repressive effects of piRNAs by degrading target mRNAs because quantitative modeling piRNA-mediated suppression of TE transcription is still challenging at this moment. However, since the piRNA-mediated transcriptional suppression of target TEs are also based on the sequence matching between piRNAs and target TEs, we expect that the evolutionary arms race signals also exist in the piRNA:TE interactions through this mechanism. More complete understanding of the TE and piRNA biology is needed to provide a thorough picture of TE:piRNA interactions in the future studies.

Many organisms have developed diverse mechanisms to repress TEs. The molecular mechanisms underlying an evolutionary arms race are important for understanding the origin and evolution of genetic and phenotypic diversities. Due to the uniqueness of piRNA biogenesis and their clearly repressive effects on TE transposition, the TE:piRNA interaction system gives us a new opportunity to detect a potentially widespread evolutionary arms race in nature. Although the TE:piRNA interaction shares similarities with the CRISPR/Cas9 system [145] in that the emergence of the suppressor elements is dependent on the invasive elements, the difference is that in the former piRNAs repress TEs by degrading mRNAs or inhibiting transcription whereas in the latter the invasive DNA fragments are destroyed. Thus, the interactions between piRNAs and TEs provide novel insights into the biology of the arms race between genomic parasites and hosts.

Understanding the population dynamics of TEs and the underlying evolutionary forces has been a research objective pursued by many evolutionary biologists [146]. Although the piRNA pathways are crucial in suppressing the activities of TEs [56], whether there is an evolutionary arms race between TEs and piRNAs was unclear [31]. In this study, we detected significantly positive Spearman’s correlations between TEs and antisense piRNAs for six TE families. Our simulations further highlight that TE activities and the strength of purifying selection against TEs are important factors shaping the interactions between TEs and piRNAs. It is possible that the piRNA repression would alleviate the deleterious effects of TEs, which causes TEs to keep increasing in the genomes. Our studies also suggest that de novo generation of piRNAs is an important mechanism to repress the newly invaded TEs. Although the interactions between TEs and piRNAs are complex and many factors should be considered to impact their interaction dynamics, our results suggest the emergence, repression specificity and strength of piRNAs on TEs should be considered in studying the landscapes of TE insertions in *Drosophila*.

## Methods

### *Drosophila* stocks and fly husbandry

The Global Diversity Lines (GDL) strains of *D. melanogaster* with whole-genome sequences were collected from five continents [110]. Genome information of 81 of these strains sequenced with Illumina 100 bp paired-end protocols was analyzed in this study. These strains were sampled from: Beijing, China (14 lines, abbreviated B); Ithaca, NY USA (17 lines, abbreviated I); Netherlands, Europe (19 lines, abbreviated N); Tasmania, Australia (17 lines, abbreviated T); and Zimbabwe, Africa (14 lines, abbreviated Z). All flies were maintained on standard yeast-cornmeal-dextrose medium at 25 °C. We chose two strains with the highest genome coverage from each population (B10, B12, I06, I17, N10, N16, T05, T07, ZW155, and ZW184) for mRNA and small RNA sequencing.

### RNA preparation and library construction

The ovaries of 3–5 day old female flies were dissected in Ringer’s solution and kept in RNAlater (Ambion) before RNA extraction. Total RNA was extracted with TRIzol reagent (Invitrogen) according to the manufacturer’s instructions. Total RNA was treated with DNaseI (Takara) before mRNA-seq library construction. The purity and concentration of RNA were validated with NanoDrop and Fragment Analyzer (AATI). The cloning of small RNAs was conducted following the procedures described previously [137]. The small RNAs of 18–30 nt were gel purified. Next, the small RNAs were subjected to ligation, reverse transcription and PCR. Sequencing was done with Illumina HiSeq-2500 sequencer (run type: single-end; read length: 50 nt).

### TE content and insertion analysis

The DNA NGS reads were filtered by trimmomatic [147]. DNA sequences were all mapped to the reference genome of *D. melanogaster* (FlyBase Release 6 or 5.57, www.FlyBase.org) with bwa [148], and mapped to TE sequences annotated in BDGP TE dataset (www.fruitfly.org) and RepBase Update (www.girinst.org/repbase) [123] with BLAT [124].

We employed two complementary approaches to identify and quantify TE polymorphism. First, for the TE insertions annotated in the reference genome of *D. melanogaster*, we only considered the 3544 TE insertions that have boundary sequences uniquely mapped to the reference genome. For the paired-end reads in each strain, we required 1) the paired-end reads to be properly mapped to the reference genome, 2) one read spanning at least 30 bp flanking one boundary site of one TE insertion, 3) the mapped sequences having no more than 4 (out of 100) mismatches (or indels) with the reference genomes, 4) the TE insertion was not detected as “Absence” in the TEMP package [111]. We employed TEMP [111] to systematically screen possible novel TE insertions in the GDL strains that were absent in the reference genome. The TE references were all the possible TE sequences from the BDGP TE dataset, Repbase Update, and FlyBase. Only the insertions by the putative functional TE and TE clusters which were filtered by 95% identity with usearch [149] were retained. The insertions located less than 100 bp away were merged. We further required the following criteria to be met in at least one strain: 1) The new insertions should have supporting evidence in both flanking sides, and 2) The frequency of insertions should exceed 80% of the total number of reads spanning the TE insertion sites. The clustering of TE copy number and TE insertions was done with Multiple Dimensional Scaling [150].

### Population parameter calculation

The SNPs of the GDL strains were obtained from Grenier et al. [110]. The population parameters *θ*_*π*_, Tajima’s *D* [116], and Fay and Wu *H* [117] were calculated from the called SNPs. SNPs were filtered if the missing value > 50% and only bi-allele SNPs were chosen. *θ*_*π*_ and Tajima’s *D* were calculated with vcftools [151]. SNP annotations were done with snpEff [152]. The genomes of *D. simulans*, *D. sechellia* and *D. yakuba* were used to find the ancestral SNP allele. The SNPs in *D. melanogaster* were converted by liftover [153]. Fay and Wu’ *H* test was calculated by Fay’s C code [117]. The composite likelihood ratio (CLR) [118–120] was calculated with a grid size of 1 (or 10) kb with SweeD [121]. Since the accurate demographic history of each local population and the global population remains unknown, we used the default parameter settings in SweeD. In each local or the global population analysis, the CLR values of SweeD were ranked for each chromosome. LD plots were plotted with Haploview [154].

### RNA expression analysis

mRNA sequences were aligned to the genome (FlyBase r5.57) with TopHat2 [155] with 2 mismatches. Gene read counts were done with HTseq-count [156]. mRNA reads were mapped to the canonical TE sequences with STAR [157]. The fold change in gene expression level induced by TE insertion is calculated from the ratio between the gene expression in the strains with TE insertion and in the strains without TE insertions.

### Small RNA analysis

We deep-sequenced small RNAs from ovaries of 10 Global Diversity Lines (GDL) strains of *D. melanogaster* and collected the ovarian small RNA-Seq data of 16 DGRP (*Drosophila* Genetic Reference Panel) strains from Song et al. [95]. For these small RNA-Seq data, the 3′-adaptor sequences were removed using the Cutadapt software [158]. The trimmed small RNA reads that are shorter than 18 nts were discarded. The small RNAs were mapped to the reference genome of *D. melanogaster* (FlyBase r5.57), the TE sequences in the BDGP TE dataset and RepBase using Bowtie2 [159]. In case a small RNA read was mapped on multiple locations, it was equally split across these locations. After removing reads mapped on rRNAs, tRNAs, miscRNAs, ncRNAs and miRNAs that were annotated in FlyBase (r5.57), the remaining small RNAs ranged from 23 to 29 nts are treated as putative piRNAs. For each strain, we normalized the 20–22 nt siRNAs that were mapped to TEs and the 23–29 nt piRNAs that were mapped on the reference genome and TEs to one million. The RPKM of piRNAs on each TE was calculated as (total weighted piRNAs on that TE)/(length of that TE) × 10^9^/(total 23–29 nt small RNA reads and 20–22 nt reads mapped to TEs). The ping-pong signals were identified with the Python script that was previously described [160].

We predicted the target of piRNAs by requiring perfect antisense matching between position 2–11 of a 23–29 nt piRNA and a TE sequence. In case a piRNA has multiple target sites, we equally split the piRNA to all the target sites. Then for each TE sequence, we calculated the weighted abundance of piRNAs that target that TE.

The de novo piRNA production signature in the flanking regions of the novel TE insertion was defined similarly as a previous study [95] and with the following requirements. (1) In the flanking 2-kb regions of the novel TE insertion, the abundance of piRNA ≥0.5 RPKM; (2) the antisense piRNAs in the upstream flanking region and the sense piRNAs in the downstream flanking region consisted of at least 70% of the total piRNAs.

### DNA copy number of TEs

We collected the Illumina paired-end DNA-Seq reads of 10 GDL and 16 DGRP strains. We mapped DNA-Seq reads to the reference genome (FlyBase r5.57) and TE sequences (a combination of FlyBase, BDGP, and RepBase) with bwa [148], respectively. We discarded the reads with only one mate mapped to the reference sequence (less than 2% on average). For each TE sequence, we calculated the coverage of DNA-Seq on each position with bedtools [161]. The median coverage values of the reads-covered sites were assigned to each TE. To exclude the potential bias caused by the different read length and sequencing depth, we also calculated the median coverage for all the autosomal single-copy genes. In each library, the median coverage for each TE was normalized by the median coverage of single-copy genes. The ratios obtained were regarded as the copy number of TEs. Note that the active part of the *P-*element (positions 819–2527, GenBank Accession number X06779) was extracted as an individual sequence and analyzed separately.

### Simulation

The forward simulations were performed following a similar approach as we previously described [99]. Briefly, the simulation begins with *N*_*e*_ (5000) diploid individuals, in which 10% of the individuals have a single TE insertion of the sample type. In each generation, two individuals were randomly selected (based on their fitness) as the parents of an offspring individual. Recombination (*r*), changing sequences to evolve into a new subtype (escaping*, e*), excision (*i*), and duplication (*d*) of TEs and piRNAs occur during meiosis. In a parent individual, a TE retrotransposes to new positions in the genome at a rate $u/\left(1+R.\frac{y_j}{x_j}\right)$, where *R* is a constant, *x*_*j*_ and *y*_*j*_ is the number of TEs and piRNAs of the same type in that individual, respectively. For each new TE insertion, it has *f* change to become a piRNA-generating locus. Only the TE that does not generate piRNAs can retrotranspose. The simulation was performed for 15,000 generations. For each parameter (or parameter combination), the whole simulation process was replicated 200 times. A simulation stops when all TE copies are purged from the population or the average fitness of the individuals is smaller than 0.05. The correlation coefficients between the copy number of TE and piRNAs of all subtypes carried in one chromosome was calculated in 1000 sampled chromosomes that have at least one TE from the populations. The correlation coefficient is not calculated when the number of individuals that have at least one TE is smaller than 1000. In case the correlation is not statistically significant in a test (*P* > 0.05), the correlation coefficient is set at 0.

## Supplementary information

**Additional file 1: Figure S1.** Tajima’s D in 10 kb bins in each population. **Figure S2.** Fay and Wu’s H in 10 kb bins in each population. **Figure S3.** The composite likelihood ratio (CLR) with a grid size of 10 kb in each population. **Figure S4.** Signatures of natural selection on the novel TE insertion located on chr3R: 15380492–15,380,496 in N population. **Figure S5.** Multidimensional scaling (MDS) of known (a) or novel (b) TE insertions across GDL strains. **Figure S6.** Percentages of reads (y-axis) that are mapped to all the sources of TE sequences with different map length and identity. **Figure S7.** Coverage of weighted piRNAs mapped to the sense (red) and anti-sense (blue) of piRNA cluster flamenco in 10 GDL strains. **Figure S8.** Coverage of weighted piRNAs mapped to the sense (red) and anti-sense (blue) of piRNA cluster 42AB in 10 GDL strains. **Figure S9.** Barplots showing the RPKMs of de novo piRNAs generated in the flanking region (2 kb) of novel TE insertions across 10 GDL strains. **Figure S10.** Barplots showing the RPKMs of de novo siRNAs generated in the flanking region (2 kb) of novel TE insertions. **Figure S11.** Longer TEs tend to be targeted by higher densities of piRNAs in the 10 GDL strains. **Figure S12.** The fitness of host organisms (left) and number of TEs carried by one chromosome when selection is weak (s = 0.5, upper) or strong (s = 20, lower). **Figure S13.** Numbers (y-axis) of TEs (blue), piTEs (pink, these are TEs that are piRNA-repressed), effective TEs (cyan) accumulated in one chromosome along the generations (x-axis) in the simulations. **Table S1.** Genome features of all novel TE insertions on all chromosomes. **Table S2.** Candidate hitchhiking events associated with TE insertions in local populations. **Table S3.** Mapping summary of sequenced small RNAs in the 10 GDL and 16 DGRP strains.

**Additional file 2: Table S4.** Statistics of correlation analysis between piRNA abundance and TE copy number in the 10 GDL and 16 DGRP strains.

## Data Availability

The datasets generated and analysed during the current study are available in the NCBI Sequence Read Archive (SRA) under accession number SRP068882, and from Song et al. [95] in NCBI SRA under accession number SRP019948.
