# Supplementary material for: The evolutionary arms race between transposable elements and piRNAs in Drosophila melanogaster
Source: BMC Evol Biol. 2020 Jan 28;20:14. doi: 10.1186/s12862-020-1580-3 (PMC6988346; doi:10.1186/s12862-020-1580-3)
Supplement: Supplementary file 1 — Additional file 1: Figure S1. Tajima’s D in 10 kb bins in each population. Figure S2. Fay and Wu’s H in 10 kb bins in each population. Figure S3. The composite likelihood ratio (CLR) with a grid size of 10 kb in each population. Figure S4. Signatures of natural selection on the novel TE insertion located on chr3R: 15380492–15,380,496 in N population. Figure S5. Multidimensional scaling (MDS) of known (a) or novel (b) TE insertions across GDL strains. Figure S6. Percentages of reads (y-axis) that are mapped to all the sources of TE sequences with different map length and identity. Figure S7. Coverage of weighted piRNAs mapped to the sense (red) and anti-sense (blue) of piRNA cluster flamenco in 10 GDL strains. Figure S8. Coverage of weighted piRNAs mapped to the sense (red) and anti-sense (blue) of piRNA cluster 42AB in 10 GDL strains. Figure S9. Barplots showing the RPKMs of de novo piRNAs generated in the flanking region (2 kb) of novel TE insertions across 10 GDL strains. Figure S10. Barplots showing the RPKMs of de novo siRNAs generated in the flanking region (2 kb) of novel TE insertions. Figure S11. Longer TEs tend to be targeted by higher densities of piRNAs in the 10 GDL strains. Figure S12. The fitness of host organisms (left) and number of TEs carried by one chromosome when selection is weak (s = 0.5, upper) or strong (s = 20, lower). Figure S13. Numbers (y-axis) of TEs (blue), piTEs (pink, these are TEs that are piRNA-repressed), effective TEs (cyan) accumulated in one chromosome along the generations (x-axis) in the simulations. Table S1. Genome features of all novel TE insertions on all chromosomes. Table S2. Candidate hitchhiking events associated with TE insertions in local populations. Table S3. Mapping summary of sequenced small RNAs in the 10 GDL and 16 DGRP strains. [file 12862_2020_1580_MOESM1_ESM.docx]

**Additional File 1:**

**
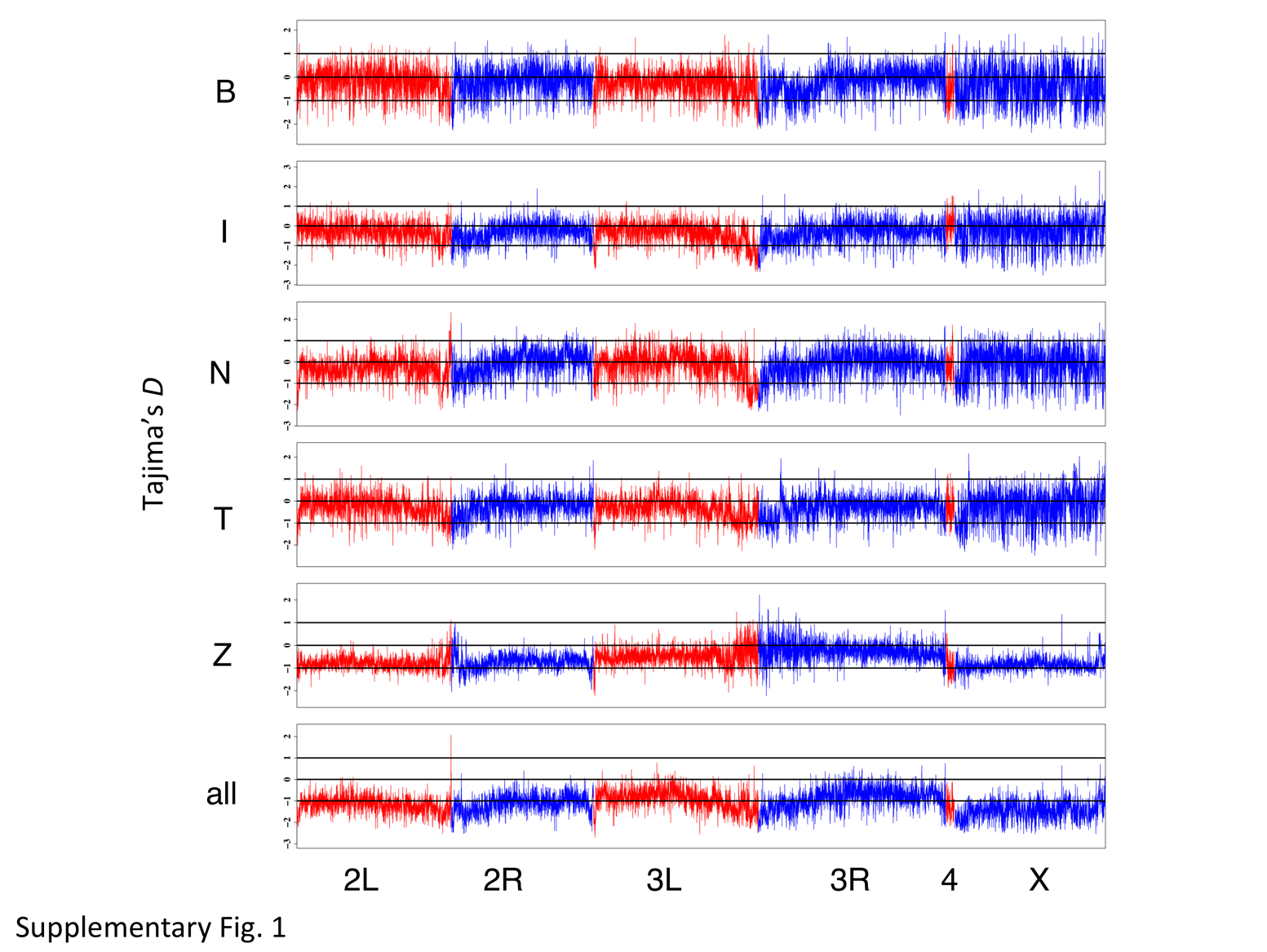
**

**Figure S1. Tajima’s *D* in 10 kb bins in each population and across all strains.**

**
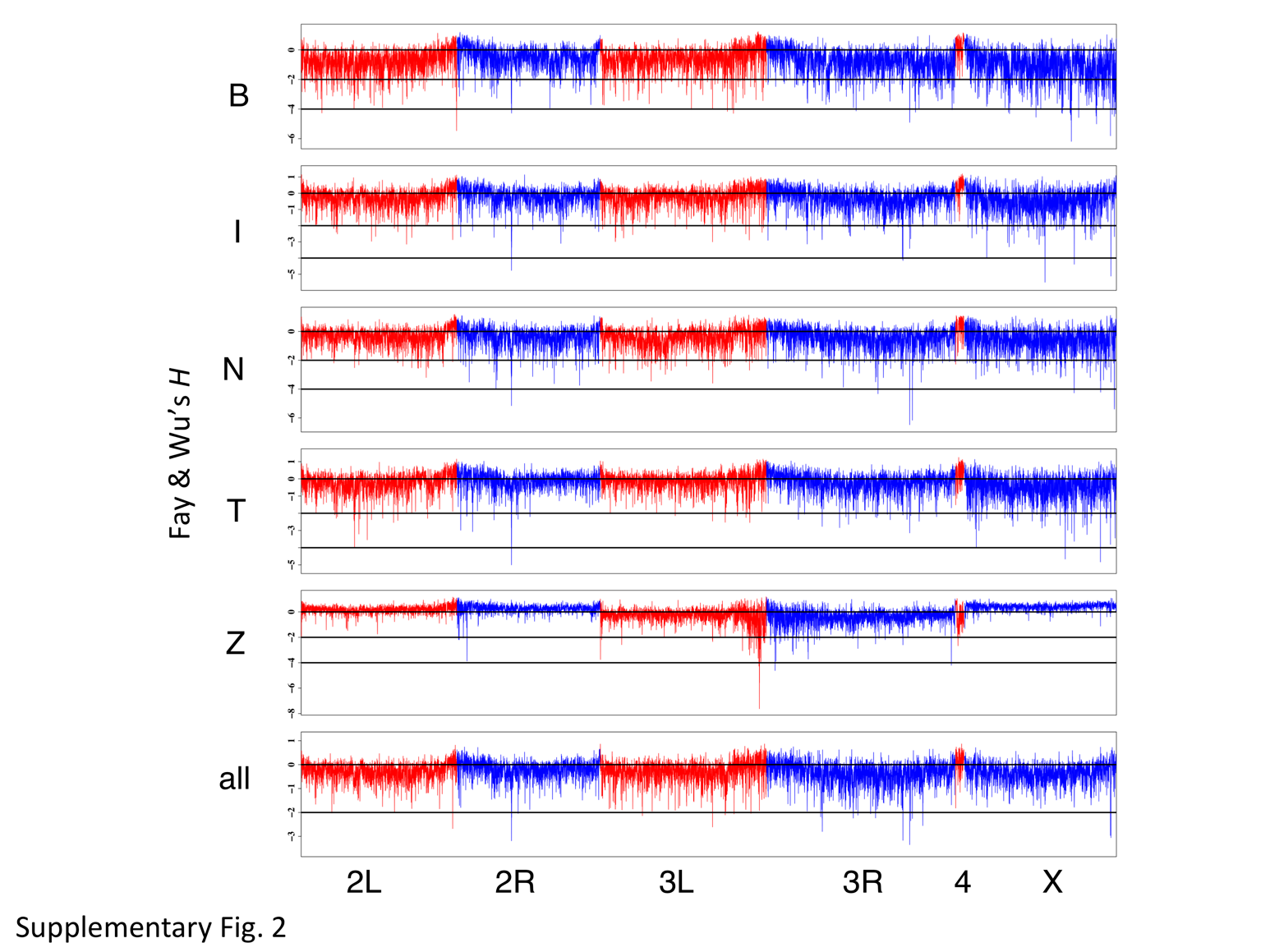
**

**Figure S2. Fay and Wu’s *H* in 10 kb bins in each population and across all strains.**

**
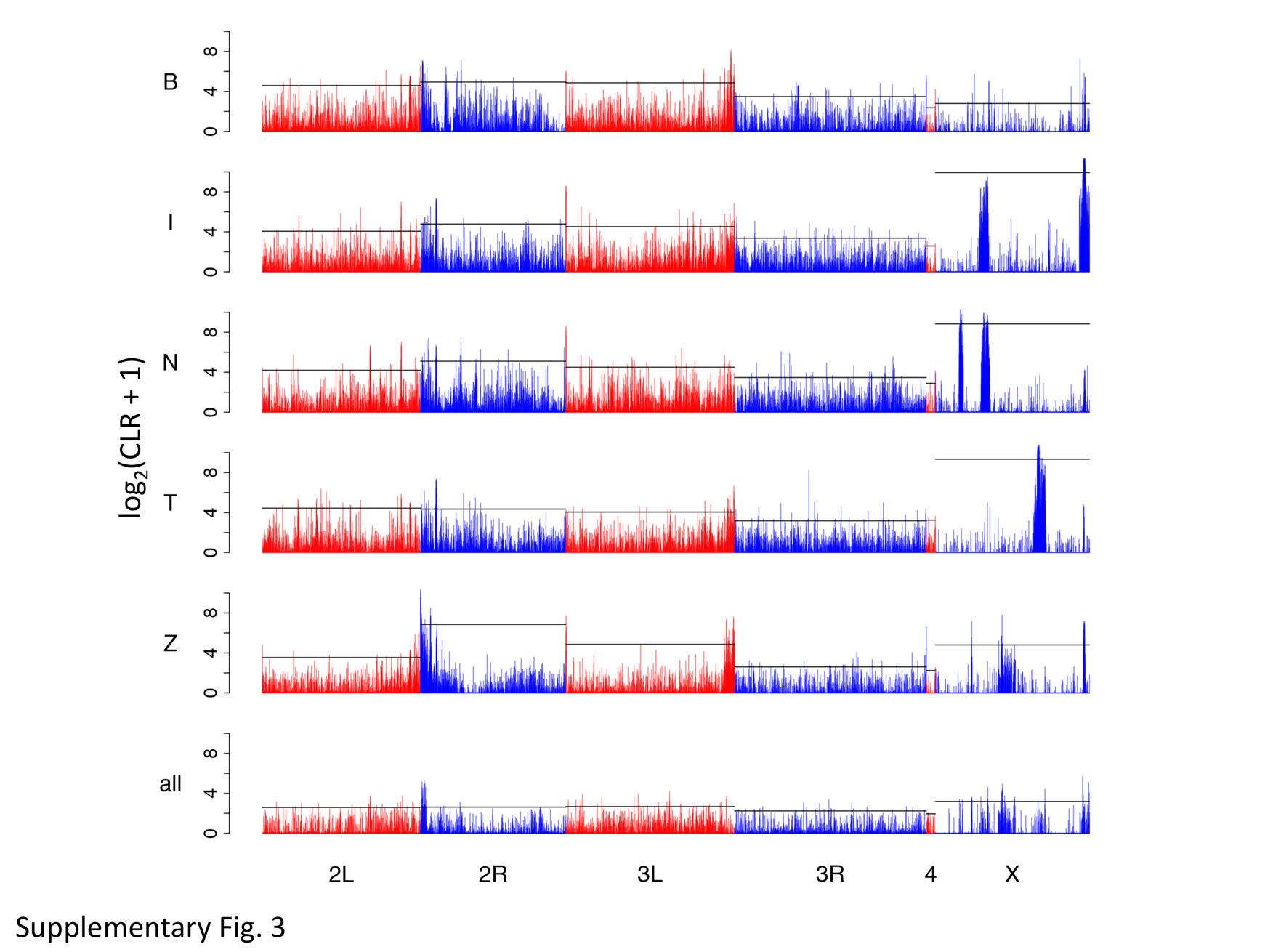
**

**Figure S3. The composite likelihood ratio (CLR) with a grid size of 10 kb in each population and across all strains.** The lines represent the 99th quantile of the CLR values on the whole chromosome.


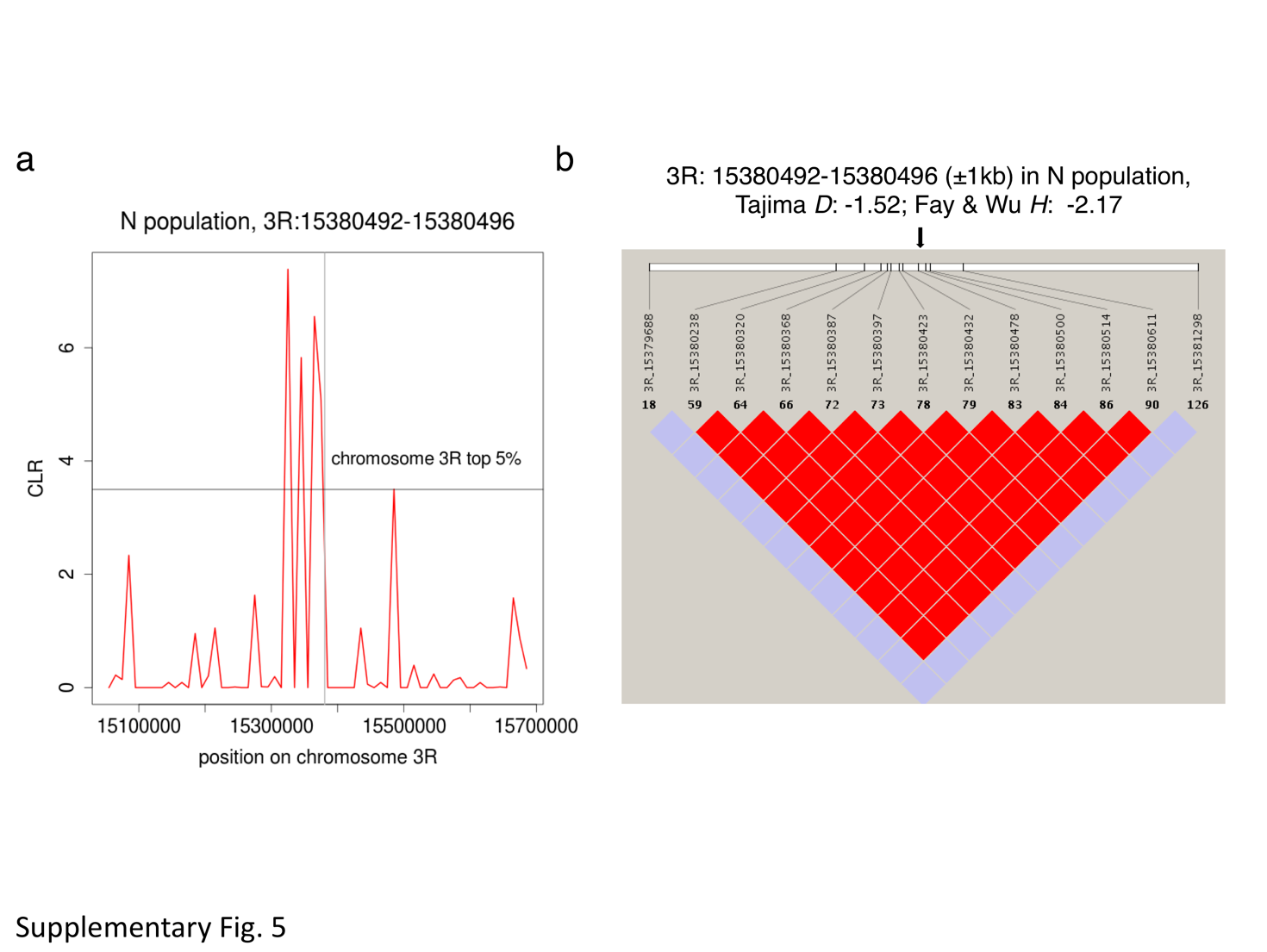


**Figure S4. Signatures of natural selection on the novel TE insertion located on chr3R: 15380492-15380496 in N population.** (a) The CLR plots for 100 kb flanking regions from SweeD analysis with a grid size of 10 kb. (b) The LD plots for 1 kb flanking regions. Coordinates are from FlyBase Release 5.


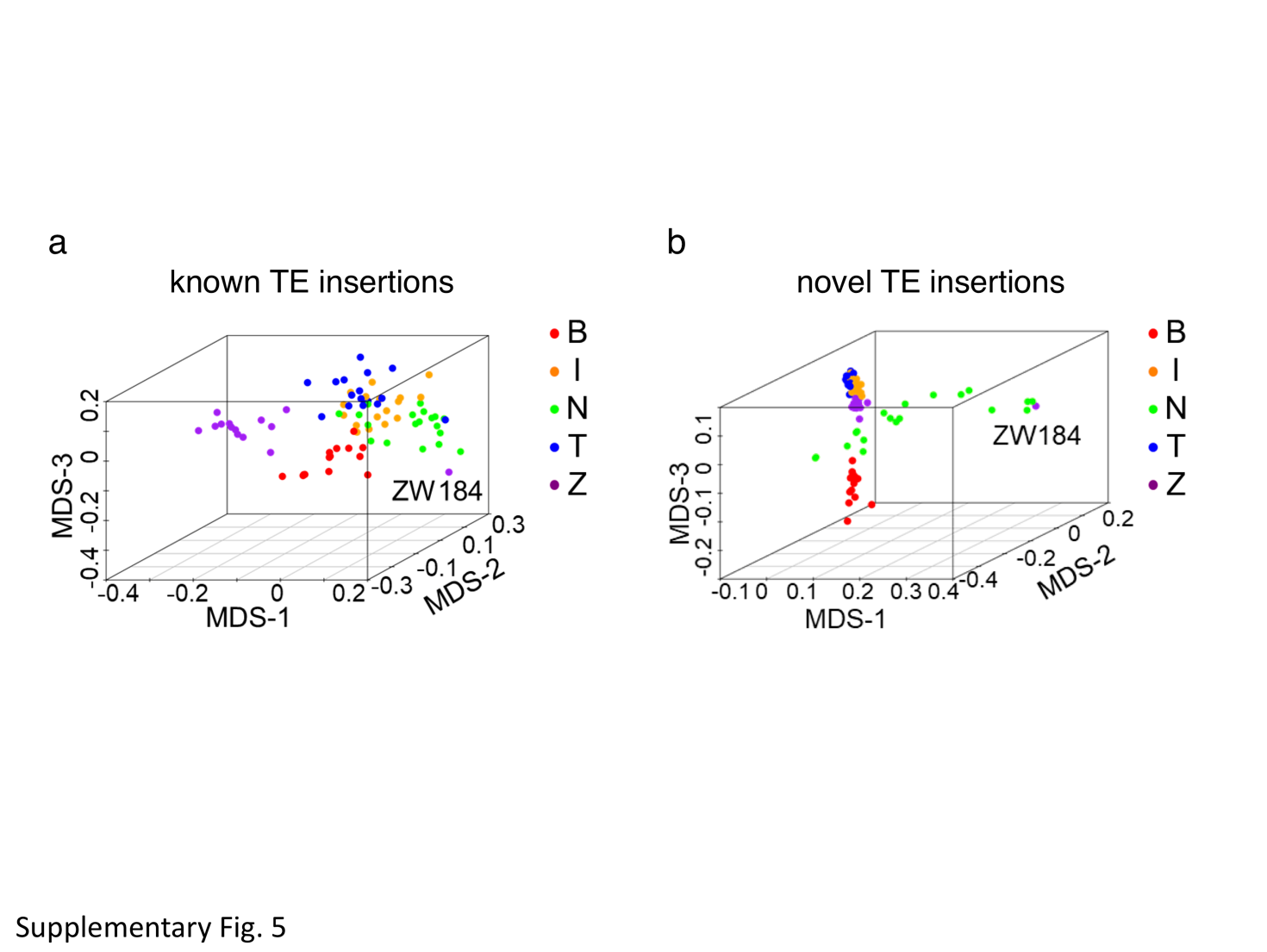


**Figure S5. Multidimensional scaling (MDS) of known (a) or novel (b) TE insertions across the GDL strains.**

**
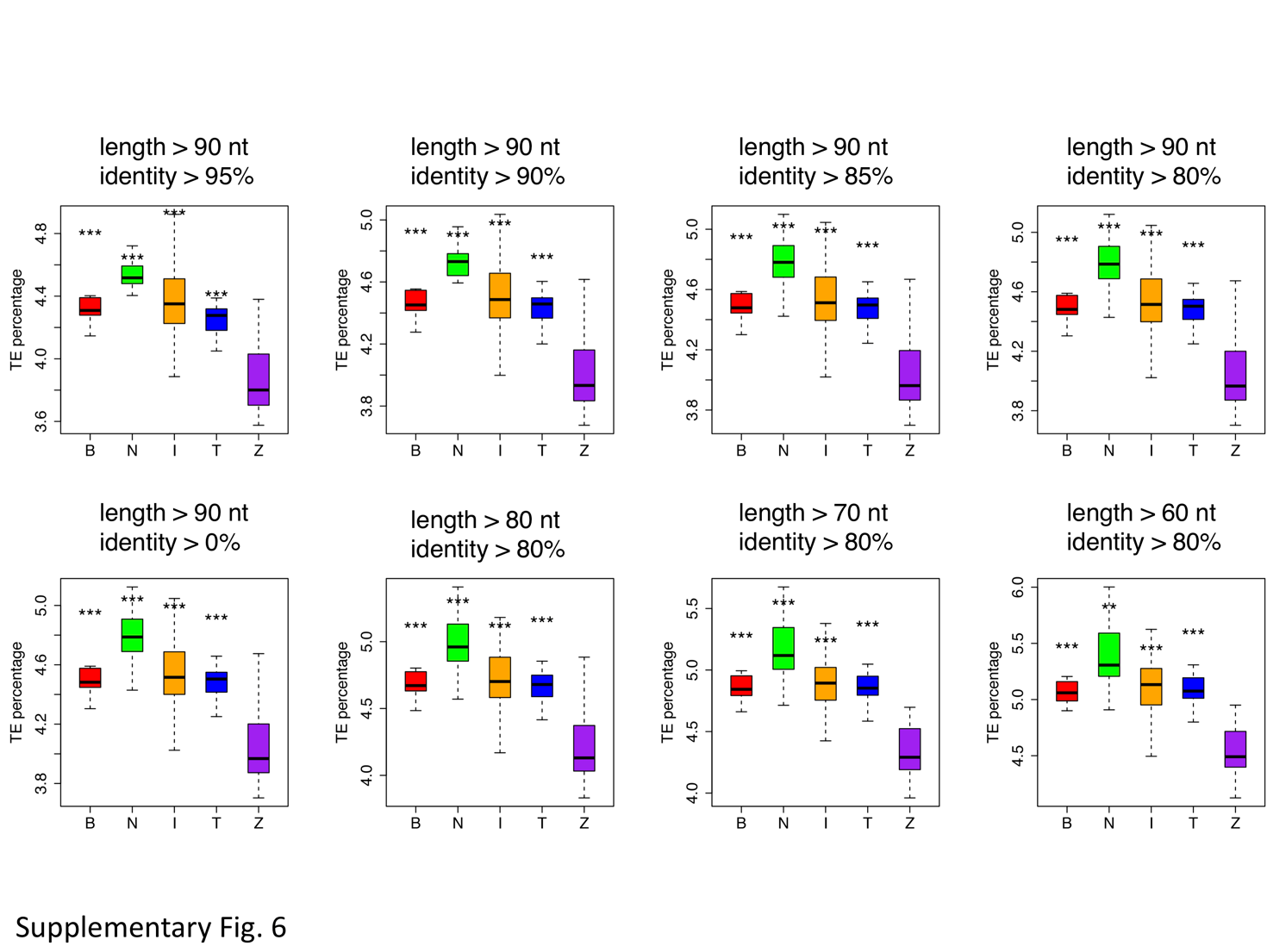
**

**Figure S6. The percentages of genomic reads (*y-*axis) that are mapped to all the sources of TE sequences across the five populations with different mapping length and identity.** The sources of TEs include reference genome, Repbase and GeneBank. KS tests were performed to test the differences in the percentages between each non-Z population and Z population. **, *P* < 0.01, ***, *P* < 0.001.

**
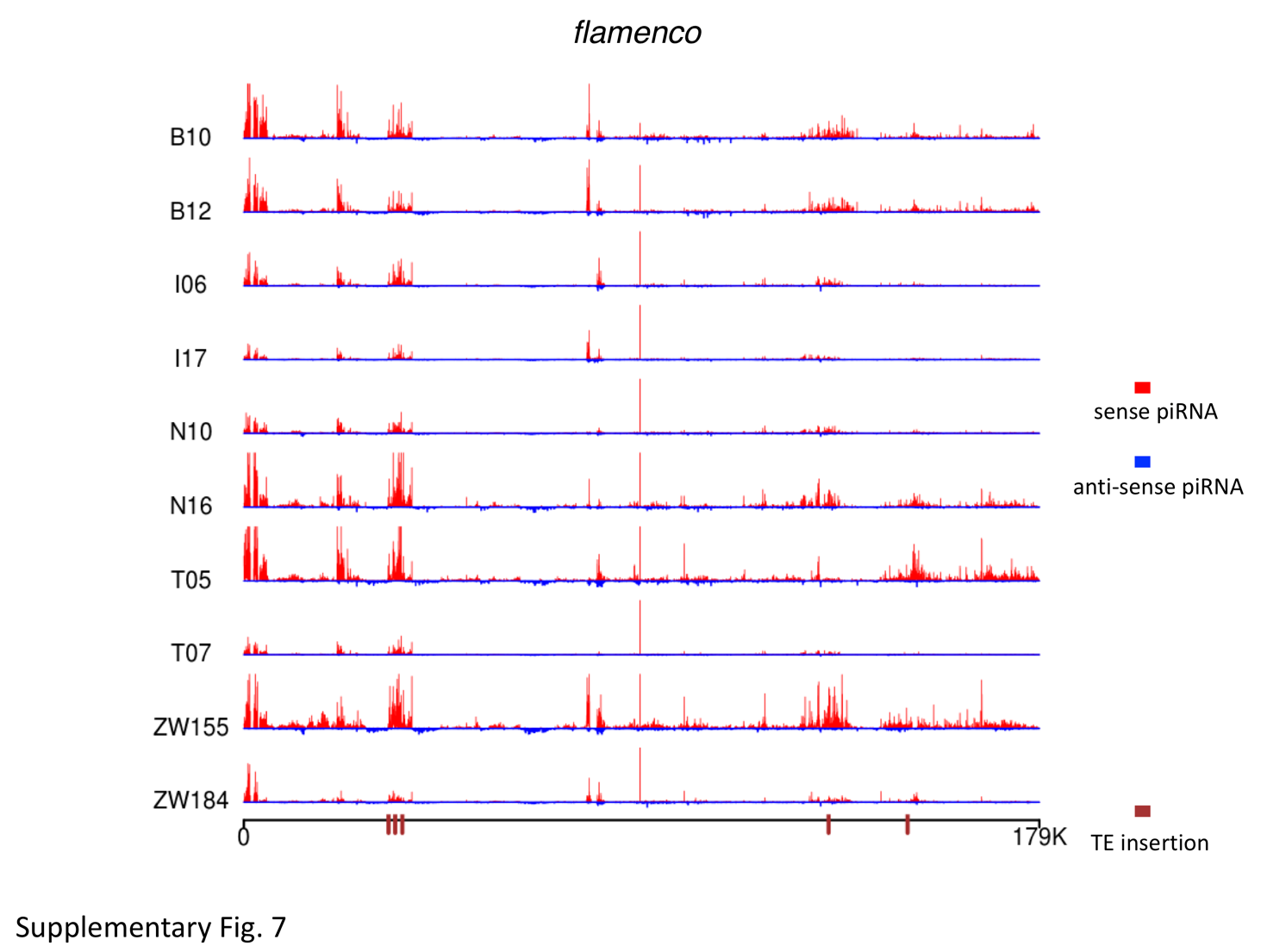
**

**Figure S7. Coverage of weighted piRNAs mapped to the sense (in red) and anti-sense (in blue) of piRNA cluster *flamenco* in 10 GDL strains.** The positions of novel TE insertion sites are displayed (in brown) on the *x*-axis.

**
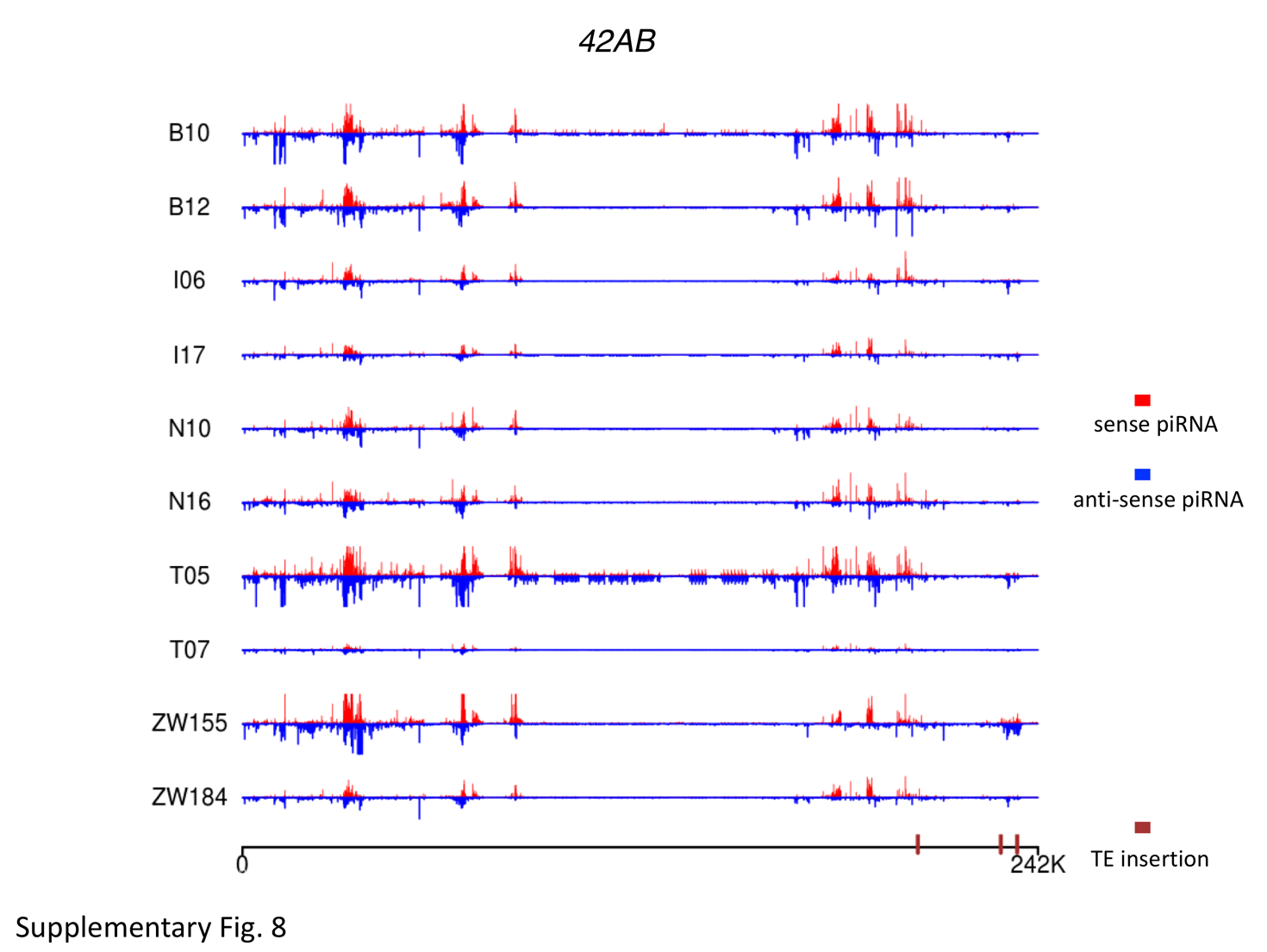
**

**Figure S8. Coverage of weighted piRNAs mapped to the sense (in red) and anti-sense (in blue) of piRNA cluster *42AB* in 10 GDL strains.** The positions of novel TE insertion sites are displayed (in brown) on the *x*-axis.


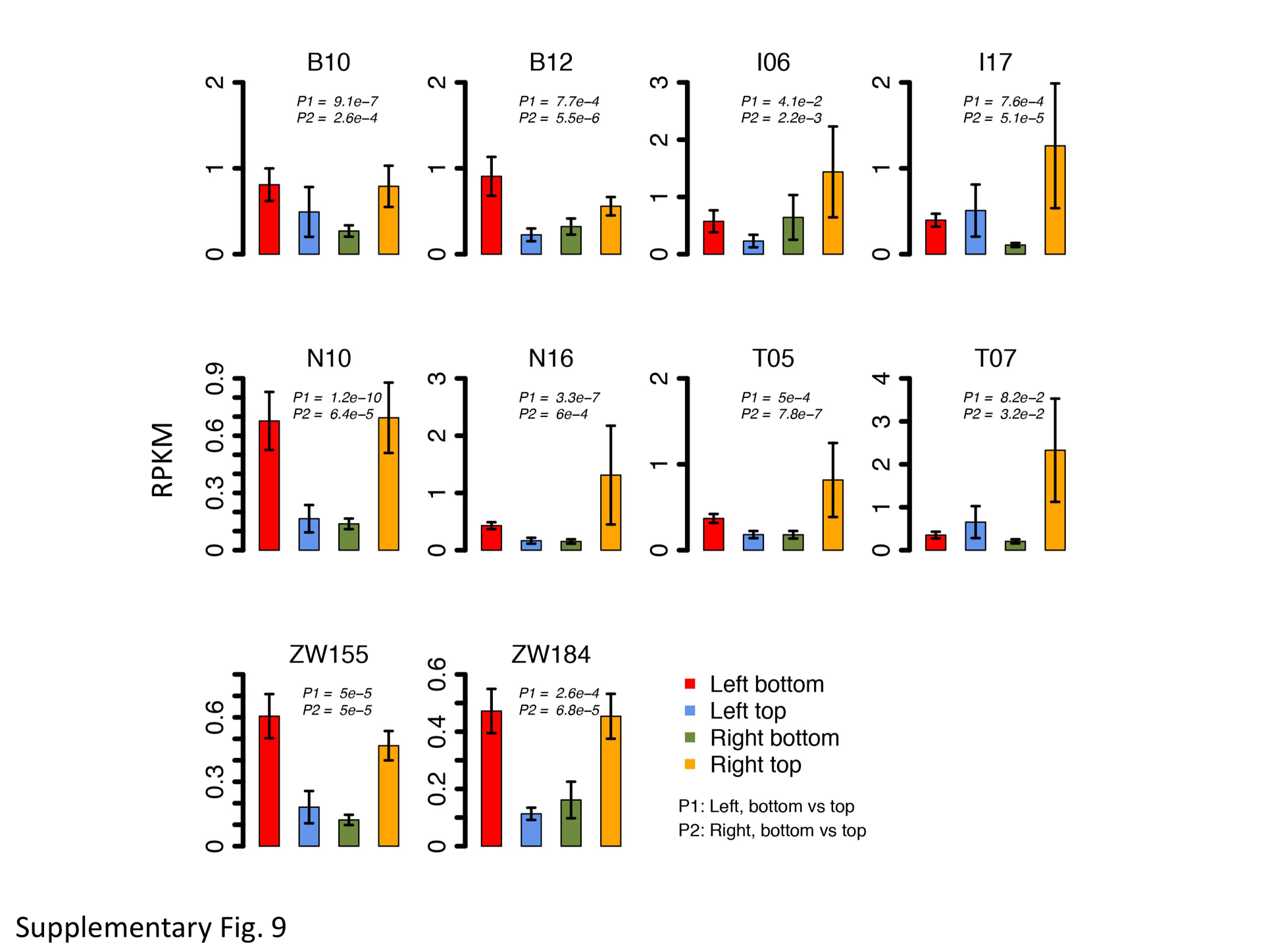


**Figure S9.** **Barplots showing the RPKMs of *de novo* piRNAs generated in the flanking region (upstream and downstream 2 kb) of novel TE insertions across 10 GDL strains.** The *de novo* piRNAs are generated with strong strand-asymmetric distributions. KS tests were performed to test the differences in the RPKM values.

**
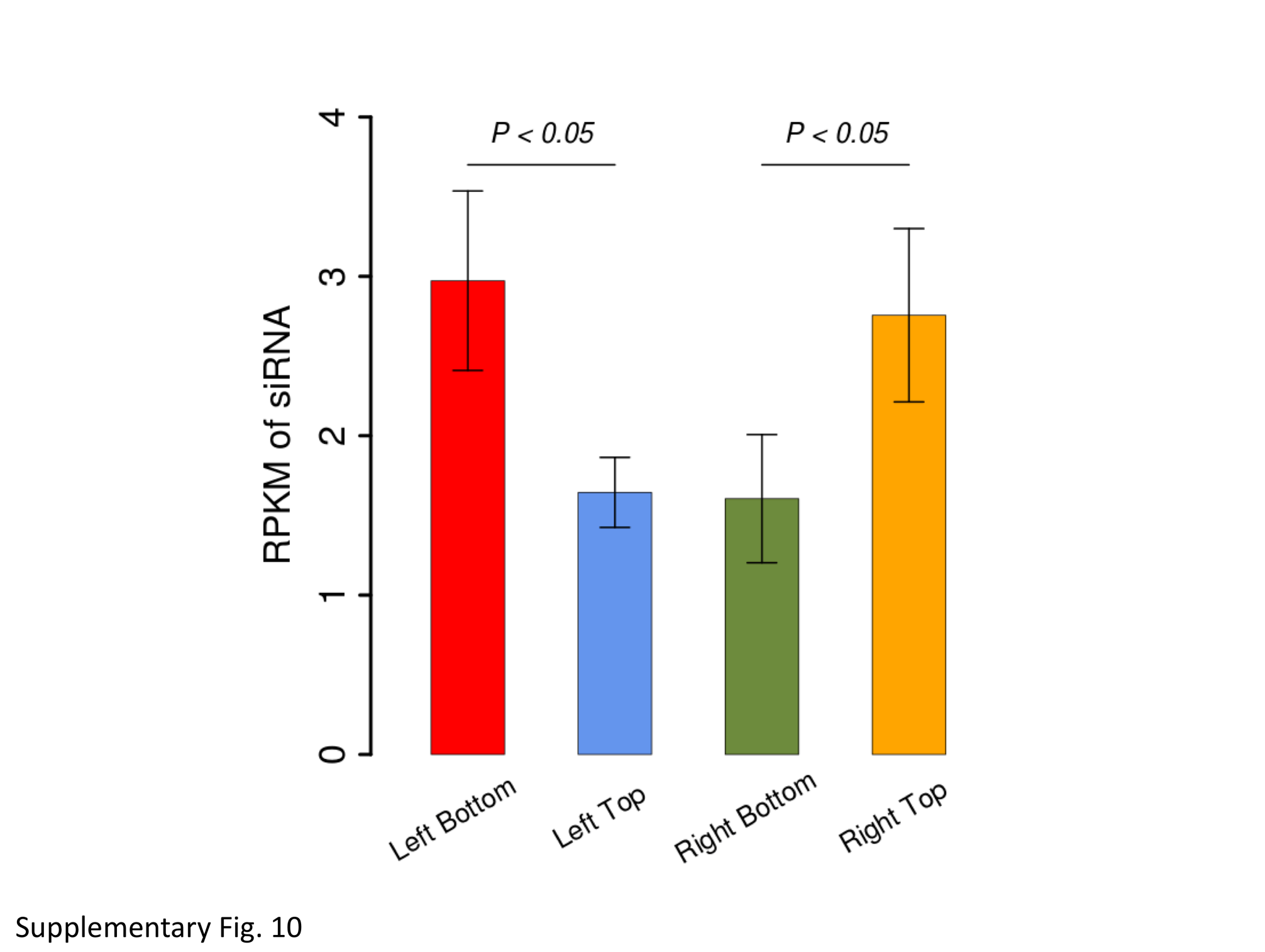
**

**Figure S10. Barplots showing the RPKMs of *de novo* siRNAs generated in the flanking region (upstream and downstream 2 Kb) of novel TE insertions.** KS tests were performed to test the differences in the RPKM values.

**
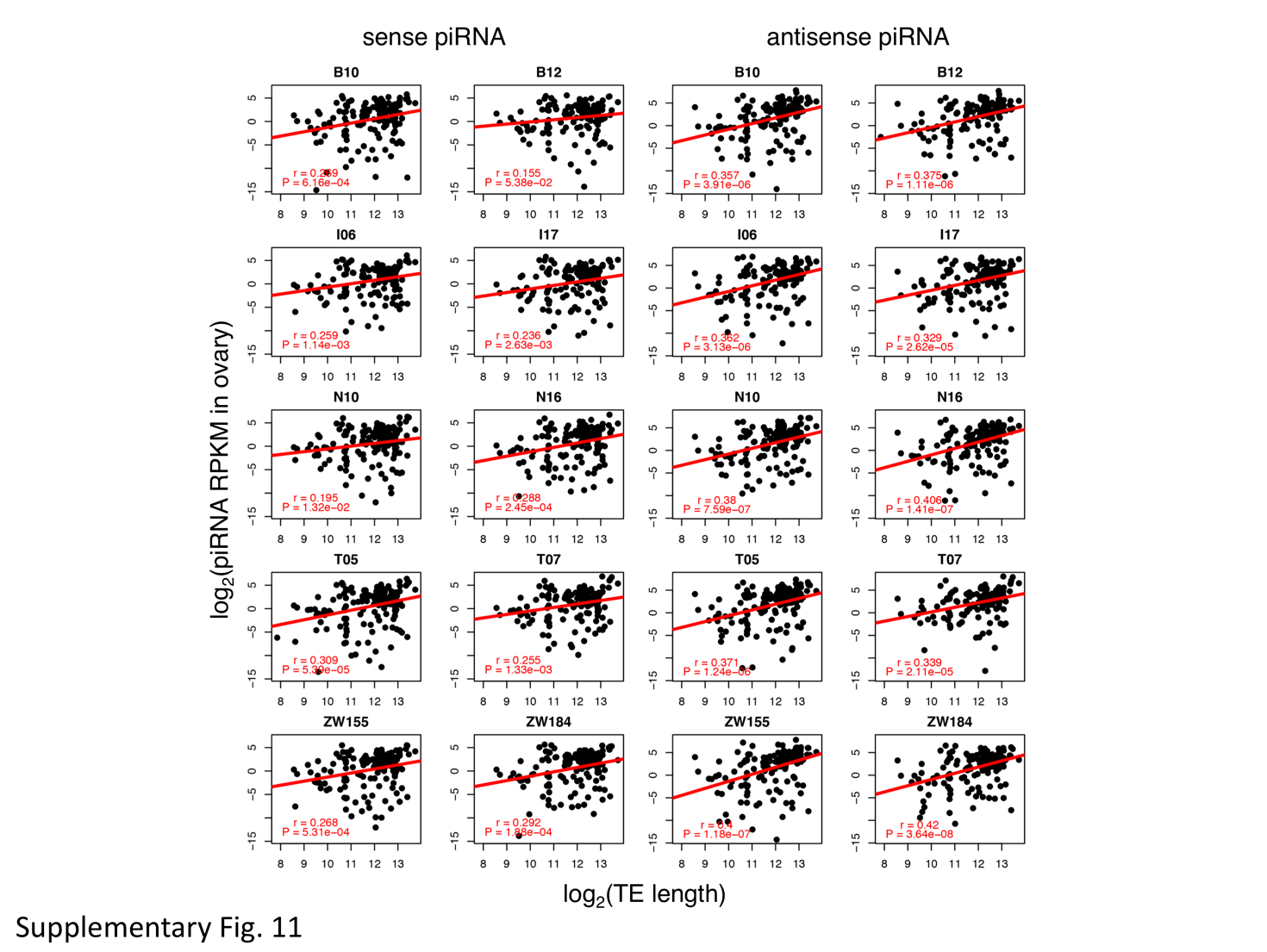
**

**Figure S11. Longer TEs tend to be targeted by higher densities of antisense and sense piRNAs in the 10 GDL strains.** The *x-*axis is the length of TEs on a log_2_ scale and *y-*axis is the log_2_(RPKM+0.1) of the piRNAs normalized matching to the reference TEs. Each dot is a TE family.

**
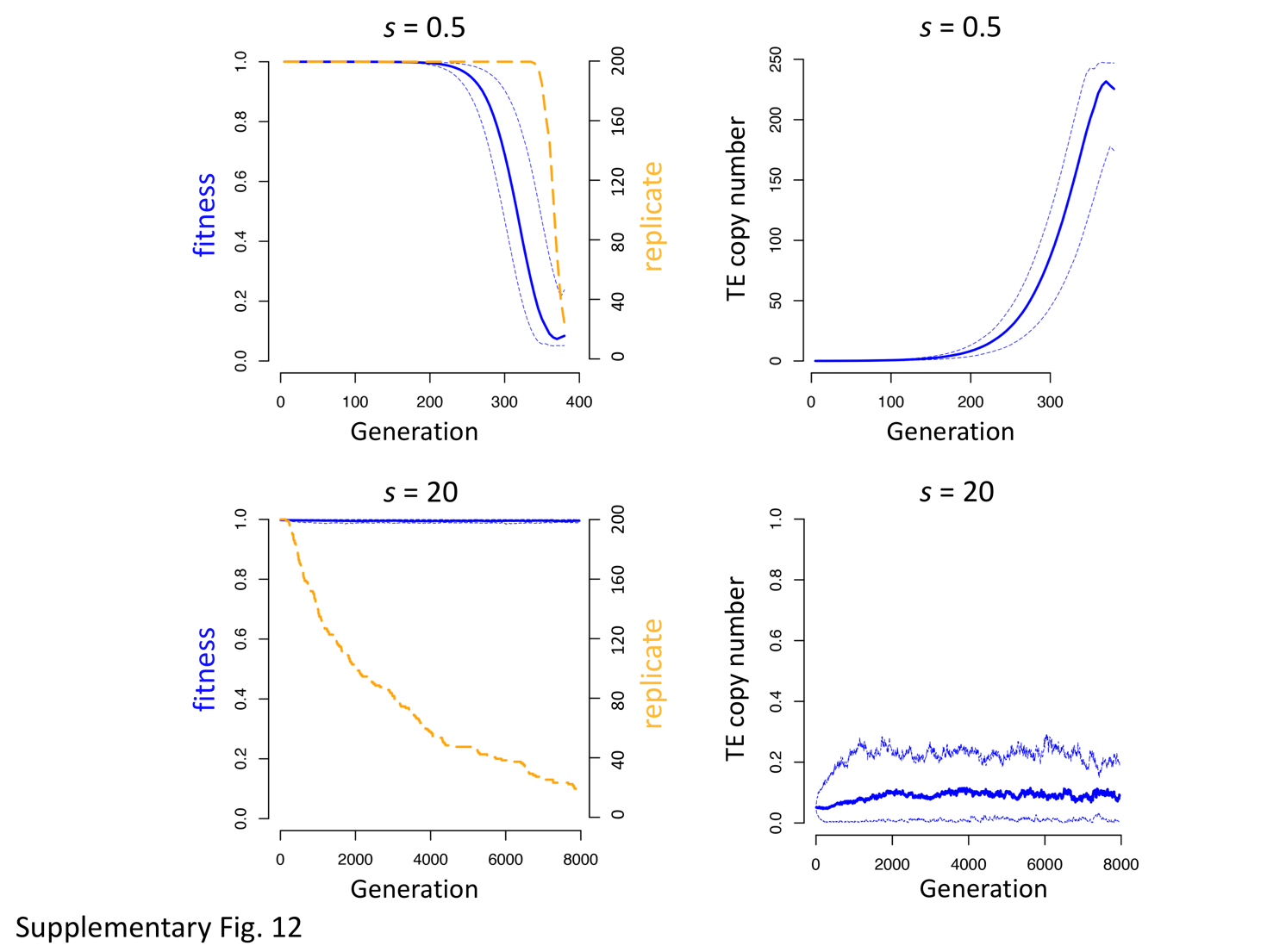
**

**Figure S12. The fitness of host organisms (left) and the number of TEs carried by one chromosome when selection is very weak (*s* = 0.5, upper) or strong (*s* = 20, lower).** Since the fitness cost of TEs has an exponential quadratic function, TEs accumulate rapidly in the population and ultimately cause extinction of the host if selection is weak (*s* = 0.5). By contrast, when selection is very strong, TEs are quickly removed from the population (*s* = 20). The mean values are given in thick blue lines and the 2.5% and 97.5% quantiles are given in thin lines. The numbers of simulation replicates (orange) along generations are also given. Each simulation replicate was ceased when the fitness fell below 0.05 (upper) or the TE is completely eliminated from the population (lower).

**
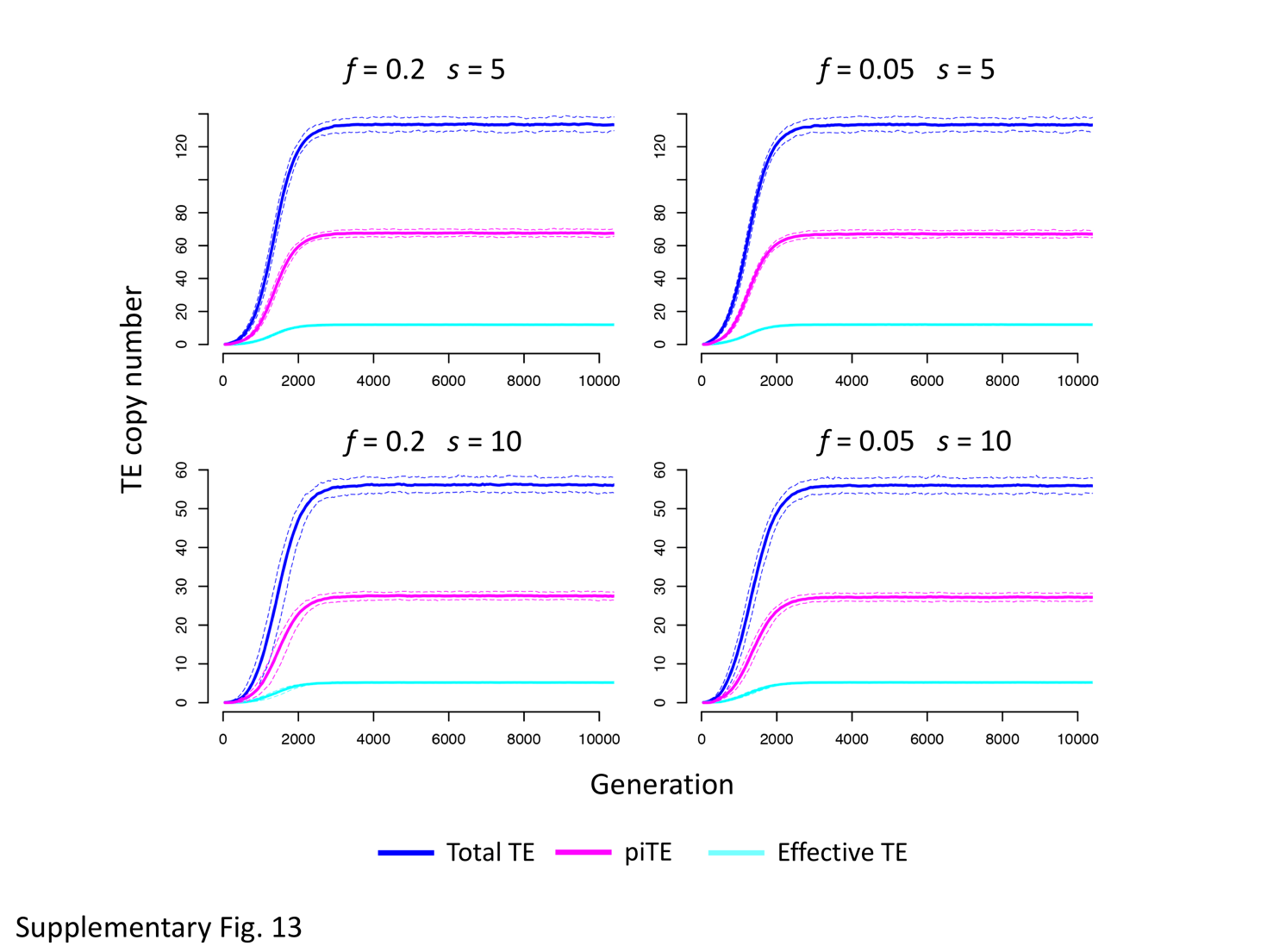
**

**Figure S13.** The numbers (*y*-axis) of TEs (blue), piTEs (pink, these are TEs that are piRNA-repressed), effective TEs (cyan) accumulated in one chromosome along the generations (*x*-axis) in the simulations. Under the same selection scaling factor (*s* = 5, upper; *s* = 10, lower), when the repressiveness of piRNAs on TEs is strong (*R* = 20), similar patterns were obtained when we set *f* = 0.2 or *f* = 0.05. *f* is the probability that a novel inserted TE is a piRNA-generating locus.

**Table S1. Genome features of all the novel TE insertions on all chromosomes.**

| **All novel TE insertions** | | | | | | | | | | |
| --- | --- | --- | --- | --- | --- | --- | --- | --- | --- | --- |
| chromosome | insertions | intron | CDS | 3'UTR | 5'UTR | ncRNA | pseudogene | stop_codon | start_codon | pre_miRNA |
| 2L | 2279 | 1238 | 47 | 54 | 115 | 54 | 5 | 3 | 0 | 1 |
| 2R | 2328 | 1276 | 56 | 86 | 147 | 52 | 4 | 0 | 2 | 0 |
| 3L | 2013 | 1091 | 39 | 44 | 106 | 58 | 1 | 0 | 0 | 0 |
| 3R | 2352 | 1241 | 50 | 45 | 150 | 65 | 4 | 5 | 0 | 0 |
| 4 | 73 | 46 | 0 | 2 | 7 | 5 | 0 | 0 | 0 | 0 |
| X | 2844 | 1887 | 22 | 52 | 142 | 240 | 2 | 0 | 1 | 2 |
| **TEs with frequency > 1** | | | | | | | | | | |
| chromosome | insertions | intron | CDS | 3'UTR | 5'UTR | ncRNA | pseudogene | stop_codon | start_codon | pre_miRNA |
| 2L | 793 | 424 | 16 | 16 | 26 | 20 | 4 | 1 | 0 | 0 |
| 2R | 854 | 439 | 17 | 25 | 53 | 21 | 3 | 0 | 1 | 0 |
| 3L | 871 | 470 | 15 | 17 | 42 | 27 | 0 | 0 | 0 | 0 |
| 3R | 1165 | 607 | 23 | 19 | 84 | 34 | 3 | 4 | 0 | 0 |
| 4 | 18 | 13 | 0 | 0 | 1 | 1 | 0 | 0 | 0 | 0 |
| X | 848 | 577 | 5 | 14 | 43 | 64 | 1 | 0 | 0 | 1 |

**Table S2. The candidate hitchhiking events associated with TE insertions in local populations.**

| Pop | Chr | Start_r5 | End_r5 | Tajima's D | Fay and Wu's H | CLR  (10kb)  quantile | CLR  (1kb)  quantile | TE  family | Freq  in global population | Freq in local population  /(strains) | Genome feature | Affected genes |
| --- | --- | --- | --- | --- | --- | --- | --- | --- | --- | --- | --- | --- |
| N | 2L | 17629100 | 17629105 | -1.19 | -1.14 | 0.59 | 0.15 | *Idefix* | 6 | 5/19 | intergenic |  |
| N | 2L | 20425367 | 20425371 | -1.18 | -1.38 | 0.61 | 0.39 | *Stalker4* | 9 | 5/19 | intergenic |  |
| N | 2R | 14414896 | 14414899 | -1.26 | -1.73 | 0.18 | 0.59 | *mdg1* | 6 | 5/19 | intergenic |  |
| B | 3L | 2244295 | 2244306 | -1.22 | -2.95 | 0.92 | 0.52 | *BS* | 7 | 6/14 | intergenic |  |
| N | 3L | 7488449 | 7488452 | -1.11 | -1.43 | 0.032 | 0.76 | *F* | 6 | 6/19 | intron | FBgn0015033 |
| N | 3L | 9342578 | 9342701 | -1.12 | -1.53 | 0.36 | 0.006 | *P-*element | 9 | 5/19 | intergenic |  |
| N | 3L | 19447804 | 19447897 | -1.33 | -1.56 | 0.60 | 0.34 | *17.6* | 9 | 5/19 | ncRNA | FBgn0266983 |
| I | 3R | 2926759 | 2926760 | -1.22 | -2.27 | 0.0061 | 0.0013 | *pogo* | 7 | 7/17 | intron | FBgn0261238 |
| B | 3R | 9145429 | 9145438 | -1.49 | -1.04 | 0.47 | 0.16 | *jockey* | 11 | 9/14 | intergenic |  |
| N | 3R | 15380492 | 15380496 | -1.52 | -2.17 | 0.030 | 0.036 | *412* | 6 | 5/19 | intron | FBgn0260003 |
| N | 3R | 27846298 | 27846369 | -1.91 | -1.83 | 0.27 | 0.13 | *roo* | 6 | 5/19 | ncRNA | FBgn0267117 |
| T | X | 370826 | 370837 | -1.11 | -1.84 | 0.86 | 0.65 | *Quasimodo* | 9 | 5/17 | intergenic |  |
| B | X | 2639225 | 2639233 | -1.06 | -1.54 | 0.20 | 0.21 | *FB* | 24 | 6/14 | intron | FBgn0028369 |
| T | X | 2639225 | 2639233 | -1.45 | -1.49 | 0.75 | 0.64 | *FB* | 24 | 9/17 | intron | FBgn0028369 |
| I | X | 4186522 | 4186526 | -1.42 | -1.76 | 0.78 | 0.46 | *Protop* | 9 | 5/17 | intergenic |  |
| N | X | 10344431 | 10344438 | -1.28 | -1.85 | 0.66 | 0.097 | *297* | 6 | 5/19 | intron | FBgn0052683 |
| N | X | 14156231 | 14156244 | -1.4 | -3.23 | 0.43 | 0.11 | *roo* | 6 | 5/19 | intron | FBgn0030558 |
| B | X | 14774580 | 14774588 | -1.58 | -2.13 | 0.16 | 0.21 | *FB* | 12 | 5/14 | intron | FBgn0264078 |
| N | X | 14774580 | 14774588 | -1.44 | 2.56 | 0.64 | 0.53 | *FB* | 12 | 6/19 | intron | FBgn0264078 |
| B | X | 18075738 | 18075741 | -1.14 | -1.61 | 0.20 | 0.21 | *pogoN1* | 11 | 10/14 | intron | FBgn0083228 |
| B | X | 18087801 | 18087806 | -1.84 | -1.63 | 0.20 | 0.21 | *hopper* | 58 | 14/14 | intron | FBgn0083228 |
| N | X | 18087801 | 18087806 | -1.26 | -2.87 | 0.12 | 0.63 | *hopper* | 58 | 17/19 | intron | FBgn0083228 |
| T | X | 18087801 | 18087806 | -1.75 | -3.24 | 0.062 | 0.39 | *hopper* | 58 | 14/17 | intron | FBgn0083228 |
| B | X | 21452429 | 21452481 | -1.46 | -2.6 | 0.20 | 0.21 | *mdg3* | 6 | 6/14 | intergenic |  |

Coordinates are from FlyBase Release 5, strains with TE insertions ≥ 5 in local populations were considered.

**Table S3. Mapping summary of sequenced small RNAs in the 10 GDL and 16 DGRP strains.**

| Strain | Reads (million) | | | | Percentage (%) | | |
| --- | --- | --- | --- | --- | --- | --- | --- |
|  | 23-29nt_total | 23-29nt_TE | 20-22nt_TE | Total | 23-29nt_total | 23-29nt_TE | 20-22nt_TE |
| B10 | 8.59 | 5.01 | 0.76 | 9.35 | 91.9 | 53.6 | 8.1 |
| B12 | 6.77 | 4.77 | 0.60 | 7.38 | 91.8 | 64.6 | 8.2 |
| I06 | 4.72 | 2.67 | 0.51 | 5.23 | 90.2 | 51.0 | 9.8 |
| I17 | 4.28 | 1.80 | 0.41 | 4.68 | 91.3 | 38.5 | 8.7 |
| N10 | 8.34 | 2.86 | 0.56 | 8.90 | 93.7 | 32.1 | 6.3 |
| N16 | 7.74 | 4.69 | 0.72 | 8.46 | 91.5 | 55.4 | 8.5 |
| T05 | 13.91 | 9.75 | 1.49 | 15.40 | 90.3 | 63.3 | 9.7 |
| T07 | 2.97 | 1.30 | 0.26 | 3.23 | 92.0 | 40.2 | 8.0 |
| ZW155 | 12.68 | 9.13 | 1.31 | 13.99 | 90.7 | 65.3 | 9.3 |
| ZW184 | 5.23 | 2.66 | 0.48 | 5.71 | 91.6 | 46.5 | 8.4 |
| DGRP313 | 13.21 | 6.87 | 0.68 | 13.90 | 95.1 | 49.4 | 4.9 |
| DGRP379 | 16.75 | 8.92 | 0.63 | 17.38 | 96.4 | 51.3 | 3.6 |
| DGRP380 | 21.01 | 11.66 | 0.73 | 21.75 | 96.6 | 53.6 | 3.4 |
| DGRP391 | 10.22 | 5.25 | 0.37 | 10.59 | 96.5 | 49.6 | 3.5 |
| DGRP358 | 19.75 | 12.14 | 1.10 | 20.86 | 94.7 | 58.2 | 5.3 |
| DGRP362 | 26.44 | 14.76 | 1.00 | 27.44 | 96.3 | 53.8 | 3.7 |
| DGRP712 | 18.33 | 9.92 | 0.75 | 19.07 | 96.1 | 52.0 | 3.9 |
| DGRP732 | 22.78 | 13.22 | 0.88 | 23.66 | 96.3 | 55.9 | 3.7 |
| DGRP375 | 26.99 | 14.11 | 0.65 | 27.64 | 97.6 | 51.0 | 2.4 |
| DGRP427 | 21.98 | 11.27 | 0.82 | 22.80 | 96.4 | 49.5 | 3.6 |
| DGRP555 | 31.95 | 17.19 | 0.76 | 32.71 | 97.7 | 52.6 | 2.3 |
| DGRP705 | 32.25 | 19.40 | 0.94 | 33.19 | 97.2 | 58.4 | 2.8 |
| DGRP707 | 18.68 | 11.12 | 0.63 | 19.31 | 96.7 | 57.6 | 3.3 |
| DGRP714 | 21.88 | 12.35 | 0.85 | 22.73 | 96.2 | 54.3 | 3.8 |
| DGRP399 | 20.89 | 12.36 | 0.89 | 21.78 | 95.9 | 56.7 | 4.1 |
| DGRP437 | 9.62 | 5.57 | 0.26 | 9.88 | 97.3 | 56.4 | 2.7 |

**Table S4. Statistics of correlation analysis between piRNA abundance and TE copy number in the 10 GDL and 16 DGRP strains. (see Additional File 2: EXCEL file Table S4.xlsx)**

“Rho” is the Spearman’s correlation coefficient between TE copy number against piRNA abundance in 10 strains, 16 strains or 26 strains, respectively.

“P” is the *P* value for Spearman’s correlation.

“P.adj” is the *P* value for Spearman's correlation adjusted after multiple testing correction.
